# Different Contribution of Redox-Sensitive Transient Receptor Potential Channels to Acetaminophen-Induced Death of Human Hepatoma Cell Line

**DOI:** 10.3389/fphar.2016.00019

**Published:** 2016-02-09

**Authors:** Heba Badr, Daisuke Kozai, Reiko Sakaguchi, Tomohiro Numata, Yasuo Mori

**Affiliations:** ^1^Laboratory of Molecular Biology, Department of Synthetic Chemistry and Biological Chemistry, Graduate School of Engineering, Kyoto UniversityKyoto, Japan; ^2^World Premier International Research Initiative-Institute for Integrated Cell-Material Sciences, Kyoto UniversityKyoto, Japan; ^3^Laboratory of Environmental Systems Biology, Department of Technology and Ecology, Hall of Global Environmental Studies, Kyoto UniversityKyoto, Japan

**Keywords:** acetaminophen, oxidative stress, TRP channels, Ca^2+^ entry, HepG2, cell death

## Abstract

Acetaminophen (APAP) is a safe analgesic antipyretic drug at prescribed doses. Its overdose, however, can cause life-threatening liver damage. Though, involvement of oxidative stress is widely acknowledged in APAP-induced hepatocellular death, the mechanism of this increased oxidative stress and the associated alterations in Ca^2+^ homeostasis are still unclear. Among members of transient receptor potential (TRP) channels activated in response to oxidative stress, we here identify that redox-sensitive TRPV1, TRPC1, TRPM2, and TRPM7 channels underlie Ca^2+^ entry and downstream cellular damages induced by APAP in human hepatoma (HepG2) cells. Our data indicate that APAP treatment of HepG2 cells resulted in increased reactive oxygen species (ROS) production, glutathione (GSH) depletion, and Ca^2+^ entry leading to increased apoptotic cell death. These responses were significantly suppressed by pretreatment with the ROS scavengers N-acetyl-L-cysteine (NAC) and 4,5-dihydroxy-1,3-benzene disulfonic acid disodium salt monohydrate (Tiron), and also by preincubation of cells with the glutathione inducer Dimethylfumarate (DMF). TRP subtype-targeted pharmacological blockers and siRNAs strategy revealed that suppression of either TRPV1, TRPC1, TRPM2, or TRPM7 reduced APAP-induced ROS formation, Ca^2+^ influx, and cell death; the effects of suppression of TRPV1 or TRPC1, known to be activated by oxidative cysteine modifications, were stronger than those of TRPM2 or TRPM7. Interestingly, TRPV1 and TRPC1 were labeled by the cysteine-selective modification reagent, 5,5′-dithiobis (2-nitrobenzoic acid)-2biotin (DTNB-2Bio), and this was attenuated by pretreatment with APAP, suggesting that APAP and/or its oxidized metabolites act directly on the modification target cysteine residues of TRPV1 and TRPC1 proteins. In human liver tissue, TRPV1, TRPC1, TRPM2, and TRPM7 channels transcripts were localized mainly to hepatocytes and Kupffer cells. Our findings strongly suggest that APAP-induced Ca^2+^ entry and subsequent hepatocellular death are regulated by multiple redox-activated cation channels, among which TRPV1 and TRPC1 play a prominent role.

## Introduction

Acetaminophen (N-acetyl-para-aminophenol, APAP) is a widely used and safe over-the-counter analgesic antipyretic drug (Rumack, 2004). However, an accidental APAP overdose can result in potentially lethal hepatotoxicity in both humans and experimental animals (Thomas, 1993; Hinson et al., 2010). Adverse reactions of APAP account for a substantial number of acute liver failure cases necessitating liver transplantation (Larson et al., 2005; Myers et al., 2007; Chun et al., 2009). Determining the exact pathways underlying APAP-induced hepatocellular death should provide important cues for preventing its lethal side effects.

It has been established that APAP overdose causes a constellation of co-related cellular events (Hinson et al., 2010). APAP is largely converted by hepatic cytochrome P450-dependent oxidases to a reactive intermediate metabolite, which depletes glutathione (GSH) and covalently binds to cellular proteins and lipids. Research on its mechanisms of toxicity has recently focused on oxidative stress exerted through accumulation of reactive oxygen species (ROS) and reactive nitrogen species (RNS), due to the depletion of GSH. APAP also causes mitochondrial dysfunction, deregulation of Ca^2+^ homeostasis, and DNA fragmentation (Muriel, 2009; Hinson et al., 2010). Furthermore, increased intracellular Ca^2+^ ([Ca^2+^]_i_) induces the mitochondrial membrane permeability transition (MPT), which promotes ROS production, loss of mitochondrial potential, cessation of ATP synthesis and eventually hepatocellular apoptosis or necrosis (Bessems and Vermeulen, 2001). Though, increased [Ca^2+^]_i_ in hepatocytes is a consequence of APAP overdose, how GSH depletion/ROS accumulation, [Ca^2+^]_i_ increases and mitochondrial dysfunction interact to induce APAP overdose-induced hepatocellular death remains elusive.

Transient receptor potential (TRP) proteins and their homologs have six putative transmembrane segments that assemble in tetramers to form Ca^2+^-permeable non-selective cation channels that regulate [Ca^2+^]_i_ levels (Clapham, 2003; Voets and Nilius, 2003). Mammalian TRP channels are classified into six subfamilies: TRPV, TRPC, TRPM, TRPA, TRPP, and TRPML and are activated by myriad extra- or intracellular physical and chemical stimuli (Numata et al., 2011). Members of the TRPV, TRPC, TRPM, and TRPA subclasses were reported to act as potential sensors of changes in cellular redox status and to contribute to ROS-induced [Ca^2+^]_i_ increases (Numata et al., 2011; Takahashi et al., 2011; Kozai et al., 2014b). Several TRP family channels have been detected in liver cells (Fonfria et al., 2006; Rychkov and Barritt, 2011). TRPC1 and TRPM7 were reported to be expressed in H4-IIE rat liver hepatoma cell lines (Brereton et al., 2001; Barritt et al., 2008), while TRPM2 channels were recently linked to APAP-induced oxidative stress and Ca^2+^ overload in mouse hepatocytes (Kheradpezhouh et al., 2014). However, expression and localization of such redox-sensitive TRP channels in human liver tissues have not been thoroughly investigated. It remains an open question how respective redox-sensitive TRP channels expressed in human hepatocytes contribute to Ca^2+^ entry and cell death induced by APAP overdose.

The goal of the present study was to profile redox-sensitive TRP channels expressed in human hepatoma cell line (HepG2), serving as a model of human liver hepatocytes, and to investigate the roles of these channels in APAP-induced oxidative stress, Ca^2+^ influx and consequent cell death. Our study included assessment of the ameliorative effects of ROS scavengers or GSH inducers on Ca^2+^ influx during APAP overdose, relevant to expression of these channels. To profile cell-specific expression of redox-sensitive TRP channels, *in situ* hybridization was used to map cellular distribution of TRP mRNAs in normal human liver tissue sections. Our results identified, for the first time, the redox-activated TRPV1, TRPC1, TRPM2, and TRPM7 channels as being critical in the mechanism of APAP-induced Ca^2+^ entry and subsequent HepG2 cell death. These channels were confirmed to be localized to human liver hepatocytes. Among these channels, functional inhibition by pharmacological agents and expression suppression by siRNA strategy revealed that the contributions of TRPV1 and TRPC1 to APAP-induced responses of HepG2 cells were bigger than those of the other TRP channels. These TRP channels might represent new therapeutic targets for reducing hepatocellular damage caused by APAP overdoses.

## Materials and methods

### Reagents

N-acetyl-para-aminophenol (APAP), capsazepine (CPZ), 2-aminoethyl diphenylborinate (2-APB), clotrimazole (CTZ), 2-(12-hydroxydodeca-5,10-diynyl)-3,5,6-trimethyl-p-benzoquinone (AA861), N-acetyl-L-cysteine (NAC), dimethylfumarate (DMF), metaphosphoric acid, triethanolamine, and cyclosporine A (CsA) were from Sigma-Aldrich (St. Louis, MO, USA). Hydrogen peroxide (H_2_O_2_) was from Wako Pure Chemical Industries (Osaka, Japan). 4,5-Dihydroxy-1,3-benzene disulfonic acid disodium salt monohydrate (tiron) was from Tokyo Kasei Kogyo chemical Co. Ltd. (Tokyo, Japan). Mitogen activated protein kinase (MAPK) inhibitors including extracellular signal-regulated kinase (ERK) inhibitor, (U0126), c-jun N-terminal kinase (JNK) inhibitor, (SP600125), and p38 kinase inhibitor, (SB203580) were from Calbiochem (La Jolla, CA, USA). N-(6-Aminohexyl)-5-chloro-2-naphthalenesulfonamide (W-7) was from Santa Cruz Biotechnology (Santa Cruz, CA, USA). Allyl isothiocyanate (AITC) was from Nacalai Tesque Inc. (Kyoto, Japan).

### cDNA cloning and recombinant plasmid construction

The plasmids of pCI-neo vector carrying human TRPV1, human TRPV2, human TRPV3, human TRPV4, mouse TRPC1, mouse TRPC4β, mouse TRPC5, human TRPM2, human TRPM7, and human TRPA1 were used as previously described (Yoshida et al., 2006; Takahashi et al., 2011). Plasmids of the pCI-neo vector carrying human TRPC1 were used as previously described (Mori et al., 2002).

### Cell culture and cDNA expression

Human embryonic kidney cell lines (HEK293, HEK293T) and HepG2 were cultured in Dulbecco's modified Eagle's medium (DMEM) (Sigma) containing 10% fetal bovine serum (FBS), 30 U/ml penicillin, and 30 μg/ml streptomycin (Meiji Seika Pharma Co., Ltd., Tokyo, Japan). Human lung fibroblast (WI-38) cells were cultured in modified Eagle's medium (MEM) containing 10% FBS, 30 U/ml penicillin, and 30 μg/ml streptomycin. All cells were grown at 37°C in a humidified atmosphere of 95% air, 5% CO_2_. HepG2 (RCB1886) and WI-38 (RCB0702) cells were purchased from RIKEN BRC (Tsukuba, Japan).

HEK293 cells were co-transfected with the recombinant plasmids and pEGFP-F (Clontech Laboratories, Palo Alto, CA, USA) as a transfection marker using SuperFect Transfection Reagent (QIAGEN, Valencia, CA, USA) according to the manufacturer's instructions. Transfected cells were grown for 36–40 h prior to performing [Ca^2+^]_i_ measurements. HEK293T cells were transfected with the recombinant plasmids using Lipofectamine 2000 transfection reagent (Invitrogen, Life Technologies Corporation, Grand Island, NY, USA) according to the manufacturer's instructions and the transfected HEK293T cells were grown for 36 h prior to performing *in situ* hybridization.

### siRNA construction

Small interfering RNA (siRNA) sequences targeting the coding regions of human TRPV1 mRNA (5′-AACCTATGTAATTCTCAC CTACATCCT-3′), human TRPC1 mRNA (5′-AAGCTTTTCTTG CTGGCGTGC-3′), human TRPM2 mRNA (5′-AAAGCCTCAGTT CGTGGATTCTT-3′), and human TRPM7 mRNA (5′-AAGAAC AAGCTATGCTTGATGCT-3′) were used. The oligonucleotide sequence used for synthesis of non-targeting siRNA is 5′-GGGTATACTAGTGAATTAG-3′ (forward) and 5′-CTAATTCACTAGTATACCC-3′ (reverse). To construct siRNA oligomers, the Silencer siRNA Construction Kit (Ambion, Life Technologies Corporation, Carlsbad, CA, USA) was used according to the manufacturer's protocol. Transfection of siRNAs at 100 nM for human TRPV1, human TRPC1, and human TRPM2 or 300 nM for human TRPM7 to HepG2 cells were carried out using Lipofectamine 2000. Cells were treated with siRNAs of human TRPV1 or human TRPC1 for 24 h and siRNAs of human TRPM2 or human TRPM7 for 48 h. They were then subjected to RT-PCR, western blotting, [Ca^2+^]_i_ measurements, intracellular ROS measurements, trypan blue exclusion assays, Hoechst33342/propidium iodide (PI) assays, caspase 3/7 activity assays, or assessment of intracellular cytochrome c levels, as indicated in the individual experiments.

### [Ca^2+^]_i_ measurements

Transfected HEK293, HepG2, and siRNA-transfected HepG2 cells were subjected to [Ca^2+^]_i_ measurements 3–16 h after plating onto poly-L-lysine-coated glass coverslips. Fura-2-AM (Dojindo, Kumamoto, Japan) fluorescence was measured in HEPES-buffered saline (HBS) containing the following: 107 mM NaCl, 6 mM KCl, 1.2 mM MgSO_4_, 2 mM CaCl_2_, 11.5 mM glucose, and 20 mM HEPES (pH was adjusted to 7.4 with NaOH). Fluorescence images of cells were recorded and analyzed with the video image analysis system AQUACOSMOS (Hamamatsu Photonics, Shizuoka, Japan) according to the manufacturer's instructions. The 340:380 nm ratio images were obtained on a pixel-by-pixel basis. Fura-2 measurements were performed at 37°C in HEPES-buffered saline. The 340:380 nm ratio images were converted to Ca^2+^ concentrations by *in vivo* calibration using 10 μM ionomycin (Calbiochem/EMD Chemicals, San Diego, CA, USA) as described previously (Takahashi et al., 2008).

### Reverse transcriptase polymerase chain reaction (RT-PCR) and PCR

Total RNA from HepG2, WI-38, and siRNA-transfected HepG2 cells was extracted using ISOGEN (Wako Pure Chemical Industries) according to the manufacturer's instructions. The concentration and purity of RNA were determined using a NanoVue Plus spectrophotometer (GE Healthcare Life Science, Chalfont, Buckinghamshire, UK). Total RNA samples (0.2 μg) were reverse-transcribed at 42°C for 30 min with Avian Myeloblastosis virus reverse transcriptase using the RNA LA PCR kit (TaKaRa-Bio, Shiga, Japan). Expression levels of TRPV1-4, TRPC1, TRPC4, TRPC5, TRPM2, TRPM7, and TRPA1 in the cDNA from HepG2 and the cDNA library of human liver (TaKaRa-Bio, Code No. 9505, Lot No. A602) were determined by PCR. TRPA1 expression in the cDNA of WI-38 cells was also determined by PCR. As a positive control, we amplified the glyceraldehyde-3-phosphate-dehydrogenase (GAPDH) sequence. Suppression of RNA expression was confirmed by RT-PCR analysis. PCR was performed with LA Taq polymerase (TaKaRa) and conducted in a thermal cycler (Gene Amp PCR System 9600, Perkin Elmer Life Sciences, Boston, MA, USA) under the following conditions: initial heating at 94°C for 2 min, followed by 32–35 cycles of denaturation at 94°C for 2 min, annealing at 55–63°C for 1 min and final extension at 72°C for 1 min. Sequences of gene-specific primers (synthesized by Sigma-Aldrich), predicated lengths of PCR products and experimental conditions are listed in Table 1.

**Table 1:** **Primer sequences used in RT-PCR experiments**.

**Genes size**	**Sense/Antisense sequence**	**Annealing temperatures (°C)/Number of the thermal cycle**		**Expected size (bp)**
		**HepG2**	**cDNA of human liver**	
TRPV1	5′-ACGCTGATTGAAGACGGGAAGA-3′	58/32	58/32	295
	5′-TGCTCTCCTGTGCGATCTTGTT-3′			
TRPV2	5′-AGCAGTGGGATGTGGTAAGCTA-3′	55/35	58/32	476
	5′-TTTGTTCAGGGGCTCCAAAACG-3′			
TRPV3	5′-CGAGGATGATTTCCGACTGT-3′	55/35	58/32	328
	5′-GGGTGCACTCTGCTTCTAGG-3′			
TRPV4	5-TGGGGTCTTTCAGCACATCATC-3′	55/32	58/32	286
	5-GAGACCACGTTGATGTAGAAGG-3′			
TRPC1	5′-CAAGATTTTGGAAAATTTCTTG-3′	63/32	58/32	371
	5′-TTTGTCTTCATGATTTGCTAT-3′			
TRPC4	5′-TCTGCAAATATCTCTGGGAAGAATGC-3′	58/32	58/32	415
	5′-AAGCTTTGTTCGTGCAAATTTCCATTC-3′			
TRPC5	5′-GTGGAGTGTGTGTCTAGTTCAG-3′	58/32	58/32	501
	5′-AGACAGCATGGGAAACAGGAAC-3′			
TRPM2	5′-CTGGGAGACGGAGTTCCTGA-3′	58/32	58/32	262
	5′-TGGGGTACAGCGTGTGGTTG-3′			
TRPM7	5′-GTCAGGTGTTTTTGTGGTCGCT-3′	58/32	58/32	216
	5′-AGCCTCACATACCTTAGCTCTG-3′			
TRPA1	5′-GACCACAATGGCTGGACAGCTT-3′	58/32	58/32	540
	5′-GTACCATTGCGTTGAGGGCTGT-3′			
GAPDH	5′-ACCACAGTCCATGCCATCAC-3′	55/32	58/32	451
	5′-TCCACCACCACCCTGTTGCTGTA-3′			

### Western blot analysis

HepG2 and siRNA-transfected HepG2 cells were lysed in ice-cold lysis RIPA buffer [50 mM Tris-HCl (pH 8.0), 150 mM NaCl, 1% NP-40, 0.1% sodium dodecyl sulfate (SDS), 0.5% sodium deoxycholate, 0.1 mM sodium orthovanadate, 1 mM phenylmethylsulfonyl fluoride, 10 μg/ml leupeptin, and 5 μg/ml aprotinin] at 4°C for 30 min. The supernatant, containing protein, was collected by centrifuging at 15,000 rpm for 20 min at 4°C. Protein concentrations of samples were determined using a Pierce BCA Protein Assay kit (Thermo Fisher Scientific, Rockford, IL, USA) and samples were fractionated by electrophoresis through 7.5% SDS-polyacrylamide gel electrophoresis (SDS-PAGE). Protein bands were then transferred onto a polyvinylidene difluoride (PVDF) membrane (Millipore Corp, Bedford, MA, USA). TRPV1, TRPC1, TRPM2, and TRPM7 were detected by western blotting using anti-TRPV1 (1:1000, Santa Cruz Biotechnology, sc-12498), anti-TRPC1 (1:1000, Alomone Laboratory, Jerusalem, Israel, ACC-010), anti-TRPM2 (1:1000; Lange et al., 2009), and anti-TRPM7 antibodies (1:1000; Hanano et al., 2004), respectively, with detection by the ECL system (Amersham Pharmacia Biotech, Piscataway, NJ). For loading normalization, α-tubulin was detected with an anti-α-tubulin antibody (1:3000, Sigma-Aldrich, T6074). Chemiluminescence was detected by the luminescent image analyzer LAS-3000 (Fuji film, Tokyo, Japan). The intensity of the protein bands observed for TRPV1, TRPC1, TRPM2, and TRPM7 was quantified and normalized to the intensity of α-tubulin bands using the ImageJ software.

### DTNB-2Bio labeling assay

The 5,5′-dithiobis (2-nitrobenzoic acid)-2biotin (DTNB-2Bio) labeling assay was performed as previously described (Yoshida et al., 2006). HEK293T cells transfected with human TRPV1-GFP, human TRPV4-GFP, human TRPC1-Flag, or vector (5 × 10^6^ cells) were washed with phosphate-buffered saline (PBS). Cell surface membranes were permeabilized with PBS containing 0.001% digitonin (Sigma) for 5 min. Cells were then collected and incubated in HBS solution with or without 20 mM APAP for 3 h at 37°C followed by 100 μM DTNB-2Bio for 40 min at room temperature. Cells were washed with HBS and lysed in RIPA buffer (pH 8.0). Cell lysates were incubated batchwise with NeutrAvidin-Plus beads (Thermo-Scientific) overnight at 4°C with constant shaking. Beads were rinsed three times with RIPA buffer by centrifugation at 15,000 rpm for 1 min. Proteins were eluted in RIPA buffer containing 50 mM dithiothreitol (DTT) for 60 min and denatured in SDS sample buffer containing 50 mM DTT for 30 min at room temperature. Proteins were analyzed by 7.5% SDS-PAGE, transferred to a PVDF membrane, and detected by western blotting with an anti-GFP (Clontech) or anti-Flag (Sigma) antibody.

### Intracellular reactive oxygen species (ROS) measurements

Intracellular ROS levels in HepG2 cells were measured using 2,7-dichlorofluorescein diacetate (DCF-DA, Sigma-Aldrich) following the manufacture's protocol. Cells were incubated with 20 μM DCF-DA dissolved in Hanks' balanced salt solution (HBSS) in the dark for 45 min at 37°C, then washed once with HBSS. After loading in this manner, cells were stimulated with APAP or H_2_O_2_, with or without inhibitors, for 3 h. Fluorescence intensity was measured using a Tecan Infinite M200 microplate reader (Tecan Group Ltd., Mannedorf, Switzerland) with excitation and emission wavelengths of 485 and 530 nm, respectively.

### Reduced glutathione (GSH) measurements

GSH content of HepG2 cells was determined using a GSH assay kit (Cayman Chemical Co., Ann Arbor, MI, USA), according to the manufacturer's instructions. This assay was performed on cells that had been treated with APAP or H_2_O_2_, with or without ROS scavengers (NAC, and tiron) or GSH inducer (DMF). Absorbance was measured at 410 nm on a 96-well plate using a Tecan Infinite M200 microplate reader. Samples and standards were assayed three times in triplicate. The concentrations of GSH were calculated from a standard curve produced using a range of known GSH concentrations.

### Trypan blue exclusion assay

Cell viability was assessed by trypan blue exclusion by counting cells after a 5 min incubation with 0.4% trypan blue (Maeno et al., 2000). Data are represented as the mean values of triplicates from each separate experiment.

### Hoechst33342 and propidium iodide (PI) cell death assays

Cell death was evaluated by staining cells with Hoechst33342 (2′- [4-ethoxyphenyl] -5- [4-methyl -1-piperazinyl] -2,5 ′-bi -1H-benzimidazole trihydrochloride trihydrate solution; Dojindo) and propidium iodide (PI; Setareh Biotech, Eugene, OR, USA). After 24 h stimulation with various test reagents, as indicated for the individual experiments, cells were incubated with Hoechst33342 (1 μg/ml) and PI (0.2 μg/ml) for 10 min at 37°C in 95% air, 5% CO_2_ to stain the nuclei and dead cells, respectively. Staining was visualized under a fluorescence microscope (Olympus IX81, Olympus, Tokyo, Japan) with MetaMorph software (Molecular Devices). The proportion of dead cells was calculated by dividing the number of PI-positive cells by the number of nuclei and this was expressed as percent PI-positive cells.

### Caspase 3/7 enzymatic activity assay

To detect caspase 3/7 activity, HepG2 cells (2 × 10^5^ cells per well) were cultured in 96-well plates. After overnight adherence, cells were stimulated with APAP or H_2_O_2_, with or without inhibitors, and caspase 3/7 activity analyzed using ApoONE Homogeneous Caspase 3/7 Assay kit (Promega, Madison, WI, USA) according to the manufacturer's instructions. Fluorescence was read at an excitation wavelength of 485 nm and emission wavelength of 527 nm using a Tecan Infinite M200 microplate reader.

### Measurements of cytochrome c release

To detect levels of cytochrome c released, HepG2 cells (1 × 10^6^ cells) were plated in a 6-cm cell culture dish. After overnight adherence, cells were stimulated with APAP or H_2_O_2_ with or without inhibitors, then harvested by scraping, washed with cold PBS and re-suspended in ice-cold cytosol extraction buffer containing 10 mM Tris pH 7.4, 100 mM NaCl, 1 mM EDTA, 1 mM EGTA, 1 mM NaF, 20 mM Na_4_P_2_O_7_, 2 mM Na_3_VO_4_, 1% Triton X-100, 10% glycerol, 0.1% SDS, 0.5% deoxycholate, and protease inhibitor (0.1 mM sodium orthovanadate, 1 mM phenylmethylsulfonyl fluoride, 10 μg/ml leupeptin, and 5 μg/ml aprotinin). The cell lysate was centrifuged at 10,000 rpm for 30 min at 4°C. The supernatant (cytosolic fraction) was collected and stored at 80°C. A cytochrome c ELISA kit (Invitrogen, Carlsbad, CA, USA) was used to estimate cytochrome c protein content in the HepG2 cell extracts according to the manufacturer's instructions. Measurements were performed in triplicate and the absorbance at 450 nm was determined with a Tecan Infinite M200 microplate reader. Cytochrome c levels were calculated from a standard curve produced with a range of known cytochrome c concentrations.

### DNA fragmentation assay

To detect DNA fragmentation, HepG2 cells (1 × 10^6^ cells) were plated on 6-cm culture plates and allowed to adhere overnight. Cells were then treated with various doses of APAP or H_2_O_2_ for 24 h, collected by scraping and centrifuged at 1000 × g for 3 min at 4°C. DNA was then isolated using the Apoptosis Ladder Detection Kit (Wako Pure Chemical Industries Inc.) according to the manufacturer's protocol. DNA fragments were electrophoretically separated on a 1.5% agarose gel in Tris-acetate EDTA buffer containing 40 mM Tris acetate and 1 mM EDTA. The bands were stained with SYBR® Green I (Molecular probes Inc., Eugene, Oregon, USA).

### *In situ* hybridization

hTRPV1 (69–428), hTRPC1 (1419–1923), hTRPM2 (3889–4151), hTRPM7 (371–1020), and hTRPA1 (474–960) cDNA fragments were amplified from the plasmids of pCI-neo vector carrying human TRPV1, human TRPC1, human TRPM2, human TRPM7, and human TRPA1 using two sets of primers, summarized in Table 2, and cloned into pGEM-T Easy vector (Promega). *In vitro* transcription was performed using the digoxigenin (DIG) RNA Labeling Mix (Roche Applied Science, Roche Diagnostics Deutschland GmbH, Mannheim, Germany) for synthesis of the sense or antisense DIG-labeled RNA probes according to the manufacturer's protocol. *In situ* hybridization of these transcripts was performed in HEK293T expressing TRP of interest or HepG2 cells according to the manufacturer's protocol. Human liver paraffin sections obtained from BioChain Institute, Inc. (San Leandro, CA, USA) were digested for 30 min at 37°C with a freshly prepared solution of proteinase K (Sigma-Aldrich) at a final concentration 5 μg/ml in 100 mM Tris-HCl/50 mM EDTA (pH 8.0). After deparaffinization and rehydration, sections were acetylated with 0.25% acetic anhydride in 0.1 M triethanolamine (pH 8.0). Hybridization was performed for 14–16 h at 50°C with 400 ng/ml antisense or sense probe in 5 × standard sodium citrate (SSC, 150 mM NaCl and 15 mM sodium citrate, pH 7.4), 0.5 M EDTA (pH 8.0), 1 × Denhardt's solution (0.02% Ficoll, 0.02% polyvinylpyrrolidone, 0.2 mg/ml RNasefree bovine serum albumin), 50 mg/ml Heparin, 10 mg/ml yeast tRNA, 10% Tween 20, 10% CHAPS, 10% dextran sulfate, and 50% formamide. Sections were washed twice in 2 × SSC with 50% formamide at 50°C for 15 min. They were then washed further at increasingly high stringencies up to a final condition of 0.2 × SSC at 50°C for 20 min. Washed slides were then incubated in a blocking reagent containing 100 mM Tris-HCl (pH 7.5), 150 mM NaCl, 1% normal goat serum (NGS), and 0.1% Triton X-100 at room temperature for 1 h. Immunohistochemical detection of the hybridized probes was performed using alkaline phosphatase-conjugated anti-DIG antibody (Roche Applied Science, Cat. No. 11093274910) diluted to 1:500 in blocking buffer for 16 h. Alkaline phosphatase activity was visualized by incubating for 16–24 h with nitroblue tetrazolium (Roche Applied Science) and 5-bromo-4-chloro-3-indolyl phosphate (Roche Applied Science) in buffer containing 100 mM Tris-HCl (pH 9.5), 50 mM MgCl_2_, 100 mM NaCl, and 1 mM levamisole (Sigma-Aldrich), protected from light. The colorimetric reaction was stopped by washing sections twice for 10 min in buffer containing 0.1 M Tris-HCl and 1 mM EDTA, pH 8.0. Sections were then counterstained with 0.02% fast green FCF (Sigma-Aldrich) and coverslips were mounted with Entellan® New (Merck Millipore, Darmstadt, Germany). All sections were analyzed with an Olympus IX81 microscope.

**Table 2:** **Primer sequences used for the synthesis of the probes for ***in situ*** hybridization technique**.

**Genes**	**Sense/Antisense sequence**
TRPV1	5′-CCCCCTGGATGGAGACCCTA-3′
	5′-CTGCAGAAGAGCAAGAAGCA-3′
TRPC1	5′-TTCTGTGGATTATTGGGATGA-3′
	5′-CAGAACAAAGCAAAGCAGGTG-3′
TRPM2	5′-CTGGGAGACGGAGTTCCTGA-3′
	5′-TGGGGTACAGCGTGTGGTTG-3′
TRPM7	5-TCCAGGATGTCAAATTTG-3′
	5-TATGAAATGGGAATGCAG-3′
TRPA1	5′-CCCCTCTGCATTGTGCTGTA-3′
	5′-AAAATGTGCCTGGACAATGG-3′

### Statistical analysis

All data are expressed as means ± SEM. We collected data for each condition from at least three independent experiments. Statistical analysis was performed with the Student's *t*-test for comparing two groups. In experiments involving more than two conditions, statistical analysis was performed with a one-way analysis of variance (ANOVA) and Bonferroni *post-hoc* analysis. A *P* < 0.05 was considered statistically significant. All statistical analyses were performed using Prism 6.02 (GraphPad Software, Inc., San Diego, CA, USA).

## Results

### [Ca^2+^]_i_ increases are evoked by acetaminophen and H_2_O_2_ in HepG2 cells

**Figure 1:**
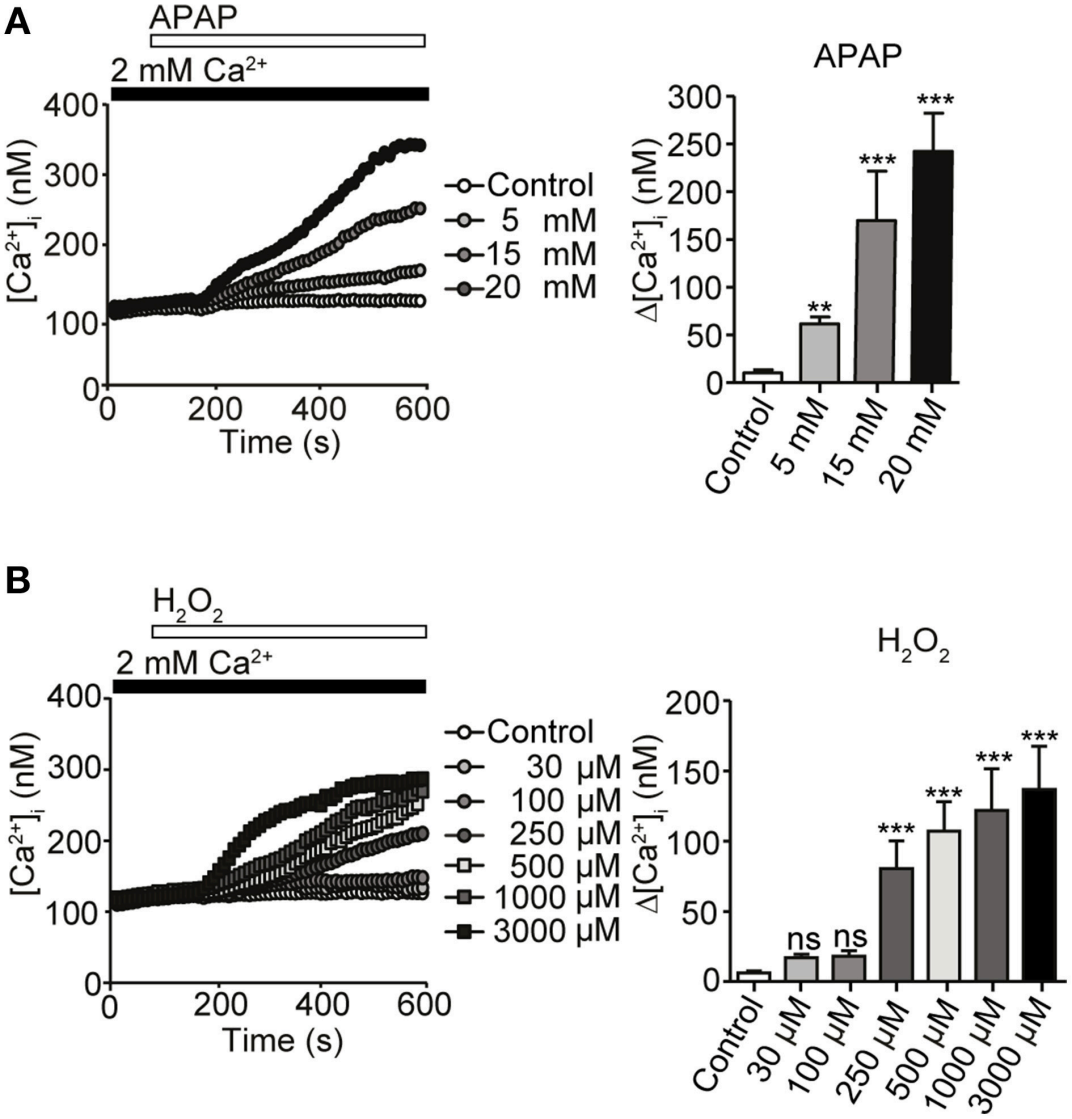
**[Ca^2+^]_i_ responses induced by acetaminophen (APAP) or H_2_O_2_ in HepG2 cells**. **(A)** [Ca^2+^]_i_ rises induced by APAP for 10 min at indicated concentrations in HepG2 cells. Average time courses (left) and maximal rise of [Ca^2+^]_i_ (Δ[Ca^2+^]_i_) (right) (*n* = 23–56). **(B)** [Ca^2+^]_i_ rises induced by H_2_O_2_ for 10 min at indicated concentrations in HepG2 cells. Average time courses (left) and Δ [Ca^2+^]_i_ (right) (*n* = 21–45). Data points are mean ± SEM. *P* ≥ 0.05, ^**^P < 0.01, and ^***^P < 0.001 compared with control. Differences not statistically significant are labeled as (ns). All data were analyzed by Student's *t*-test.

Intracellular signaling molecules such as Ca^2+^ and ROS normally regulate diverse cellular functions (Yan et al., 2006). Nonetheless, ROS-induced oxidative stress and impaired Ca^2+^ homeostasis could potentially contribute to hepatocellular death induced by APAP overdose (Hinson et al., 2010). We first tested whether APAP and H_2_O_2_ administration induced Ca^2+^ entry in HepG2 cells, using these cells as a model of human hepatocytes. Treatment of HepG2 cells with APAP at various concentrations (5, 15, or 20 mM) for 10 min resulted in a gradual, dose-dependent increase in [Ca^2+^]_i_ (Figure 1A), whereas H_2_O_2_ administration, at concentrations >100 μM, evoked abrupt [Ca^2+^]_i_ increases at 37°C (Figure 1B). Thus, Ca^2+^ entry positively correlates with the dose of APAP and ROS used for stimulation.

### APAP-evoked ROS production elicits Ca^2+^ responses in hepG2 cells

**Figure 2:**
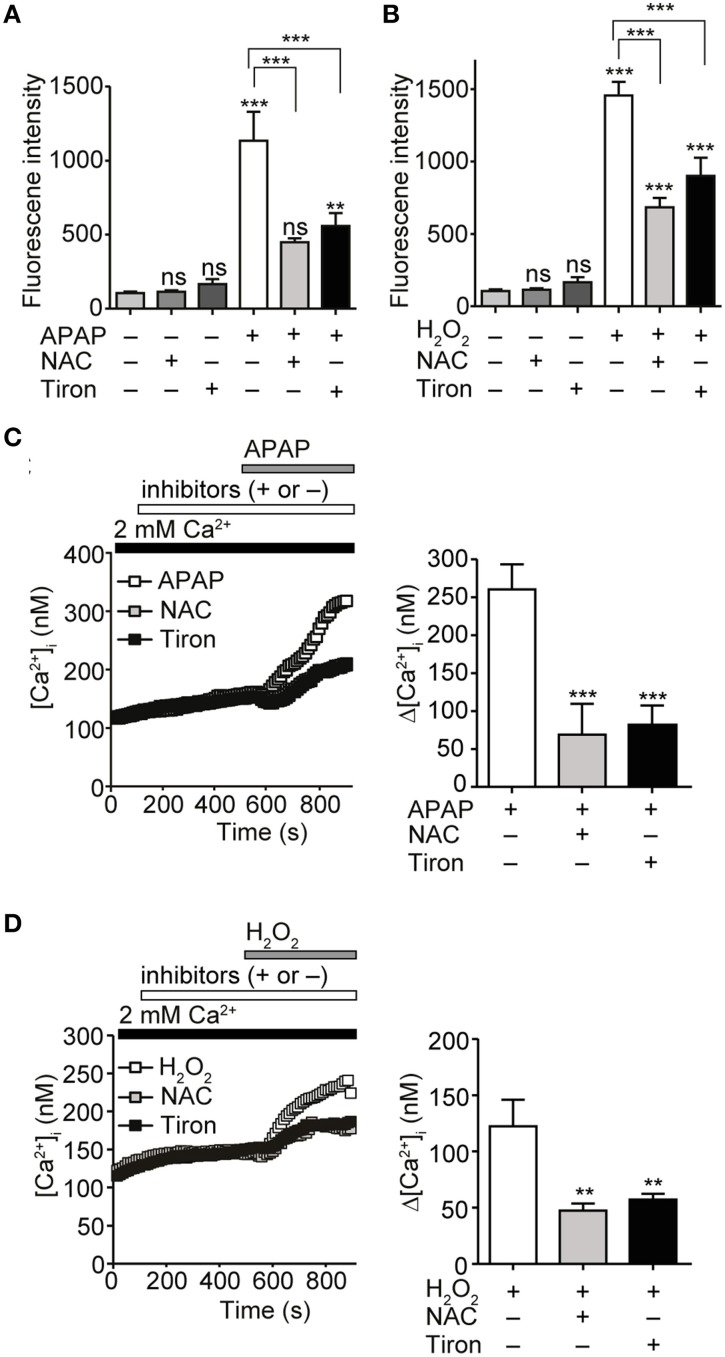
**Attenuation of APAP- or H_2_O_2_-induced ROS production and Ca^2+^ responses by ROS scavengers in HepG2 cells**. **(A)** Suppression of ROS levels induced by APAP (20 mM) treatment for 3 h by either N-acetyl-L-cysteine (NAC) or tiron (1 mM). **(B)** Suppression of ROS levels induced by H_2_O_2_ treatment (1 mM) for 3 h by either NAC or tiron (1 mM). **(C)** Effects of either NAC or tiron (1 mM) on APAP (20 mM) induced-[Ca^2+^]_i_ responses in HepG2 cells. Average time courses (left) and Δ[Ca^2+^]_i_ (right) (*n* = 24–62). **(D)** Effects of either NAC or tiron (1 mM) on H_2_O_2_ (1mM) induced-Ca^2+^ entry in HepG2 cells. Average time courses (left) and Δ[Ca^2+^]_i_ (right) (*n* = 19–33). ROS scavengers are applied for 3 h before and during APAP or H_2_O_2_ stimulation. Data points are mean ± SEM. *P* ≥ 0.05, ^**^P < 0.01, and ^***^P < 0.001. Differences not statistically significant are labeled as (ns). All data of [Ca^2+^]_i_ measurements were analyzed by Student's *t*-test, while those of ROS measurements were analyzed by ANOVA and Bonferroni *post-hoc*.

APAP induces ROS generation (DeVries, 1981). We confirmed that treatment with 20 mM APAP or 1 mM H_2_O_2_ for 3 h significantly increased ROS levels in HepG2 cells, as compared with in untreated controls. Pretreatment for 3 h with the ROS scavengers N-acetyl-L-cysteine (NAC) and tiron, each at 1 mM, significantly reduced ROS levels produced after treatment with either 20 mM APAP or 1 mM H_2_O_2_ for additional 3 h (Figures 2A,B). Administration of either of the ROS scavengers alone did not significantly change ROS levels, as compared with those in untreated control cells. In [Ca^2+^]_i_ measurements, increases in [Ca^2+^]_i_ in response to 20 mM of APAP or 1 mM H_2_O_2_ were also significantly suppressed by pretreatment with either NAC or tiron, at 1 mM, for 6 min (Figures 2C,D). Together, these results indicate that ROS production evoked by APAP and H_2_O_2_ induces the Ca^2+^ responses in HepG2 cells.

### Characterization of redox-sensitive TRP channels found in HepG2 using HEK293 cell system

**Figure 3:**
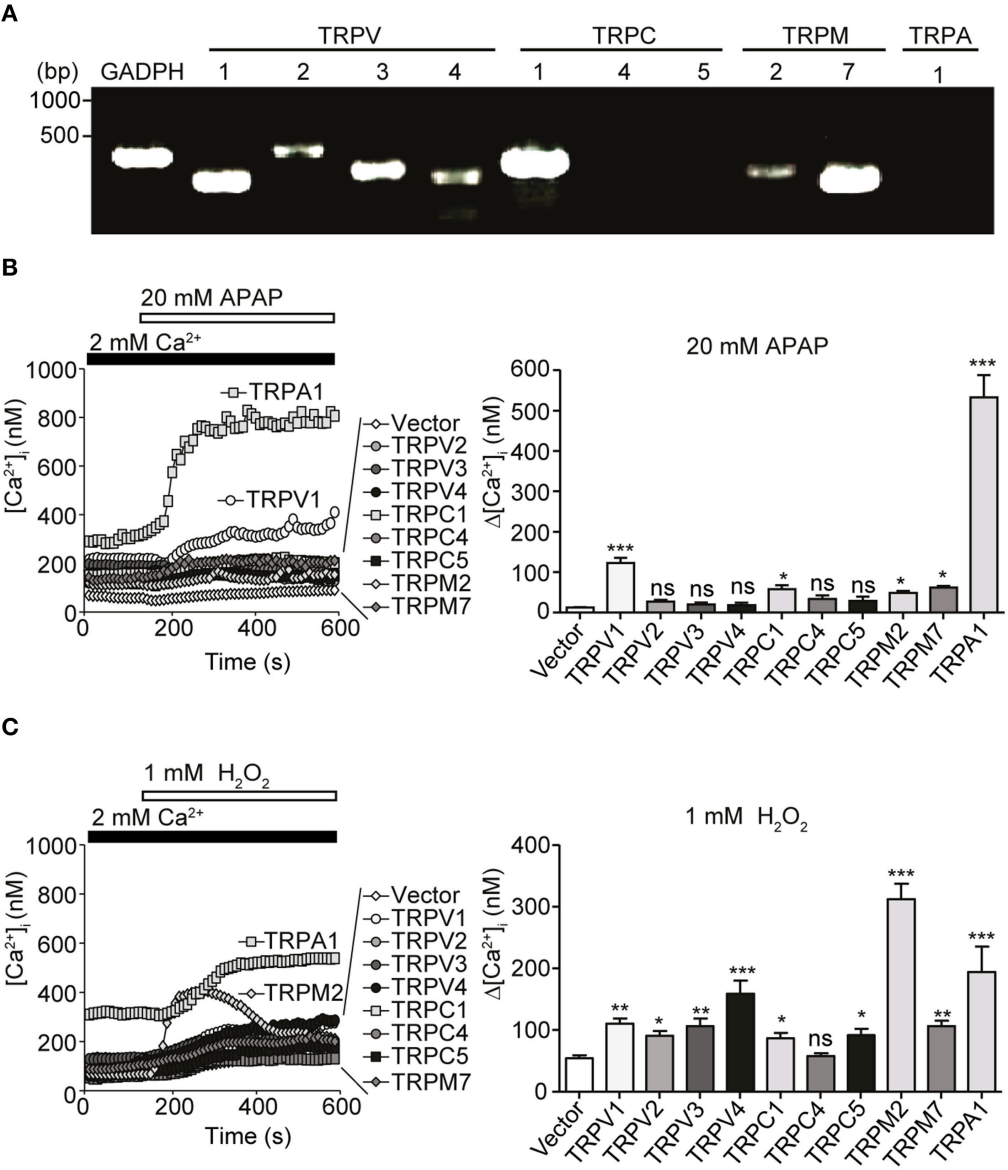
**Expression of redox-sensitive TRP channels in HepG2 cells and their responses when expressed in HEK293 cells**. **(A)** Expression of redox-sensitive TRP channel mRNAs [TRPV1-4, TRPC1, TRPC4, TRPC5, TRPM2, TRPM7, TRPA1, and glyceraldehyde-3-phosphate dehydrogenase (GAPDH)] detected by RT-PCR in total RNA isolated from HepG2 cells. Specific PCR primers used are listed in Table 1. **(B,C)** [Ca^2+^]_i_ responsesevoked by 20 mM APAP **(B)** or 1 mM H_2_O_2_ **(C)** in HEK293 cells expressing human TRPV1, human TRPV2, human TRPV3, human TRPV4, mouse TRPC1, mouse TRPC4β, mouse TRPC5, human TRPM2, human TRPM7, human TRPA1, or vector. Average time courses (left) and Δ[Ca^2+^]_i_ (right) (*n* = 16–64). Data points are mean ± SEM. *P*≥0.05, ^*^P < 0.05, ^**^P < 0.01, and ^***^P < 0.001 compared to vector. Differences not statistically significant are labeled as (ns). All data were analyzed by Student's *t*-test.

The data above suggested a role for ROS in mediating [Ca^2+^]_i_ increases in response to APAP overdose in HepG2 cells. TRPV1, TRPV3, TRPV4, TRPC1, TRPC4, TRPC5 (Yoshida et al., 2006), TRPM2 (Hara et al., 2002), TRPM7 (Aarts et al., 2003), and TRPA1 channels (Takahashi et al., 2008) were reportedly activated by H_2_O_2_. Therefore, we hypothesized that these channels might be involved in the aforementioned APAP-induced Ca^2+^ responses in HepG2 cells. To investigate this, we first examined expression of mRNAs for redox-sensitive TRPV1-4, TRPC1, TRPC4, TRPC5, TRPM2, TRPM7, and TRPA1 channels by RT-PCR in HepG2 cells. As shown in Figure 3A, RNAs encoding TRPV1-4, TRPC1, TRPM2, and TRPM7 were detected in HepG2 cells. Next, we tested whether 20 mM APAP activates recombinant ROS-sensitive TRP channels expressed in HEK293 cells. APAP, at 20 mM, evoked [Ca^2+^]_i_ responses in HEK293 cells expressing TRPV1, TRPC1, TRPM2, TRPM7, or TRPA1 but not in cells expressing TRPV2, TRPV3, TRPV4, TRPC4, or TRPC5 (Figure 3B). In contrast, all TRP channels except TRPC4 responded to H_2_O_2_ (Figure 3C), as previously reported (Hara et al., 2002; Aarts et al., 2003; Yoshida et al., 2006; Takahashi et al., 2008). These results illustrate the sensitivity of HEK293 cells expressing TRPV1, TRPC1, TRPM2, TRPM7, and TRPA1 to both APAP and H_2_O_2_. Though, TRPA1, among all the channels, showed the highest Ca^2+^ responses to APAP, its molecular expression had not, as noted above, been detected in HepG2 cells. This was confirmed by comparing HepG2 with a positive control, demonstrating TRPA1 mRNA expression in WI-38 human lung fibroblast cells (Supplementary Figure 1A), which express functional TRPA1 (Jaquemar et al., 1999; Hu et al., 2010; Kozai et al., 2014a). In addition, we found that 100 μM AITC, a TRPA1 agonist (Jordt et al., 2004), failed to elicit a Ca^2+^ response in HepG2 cells (Supplementary Figure 1B). Therefore, TRPV1 is the most responsive to APAP, among the recombinant TRPs that are found being expressed in HepG2 cells. Overall, these results raise a possibility that redox-sensitive TRPV1, TRPC1, TRPM2, and TRPM7 contribute to the APAP-induced Ca^2+^ responses in HepG2 cells.

### APAP-evoked Ca^2+^ entry and ROS production in HepG2 cells are mediated by TRPV1, TRPC1, TRPM2, and TRPM7

To characterize involvement of endogenous redox-sensitive TRPV1, TRPC1, TRPM2, and TRPM7 in the APAP-induced Ca^2+^ responses in HepG2 cells, we employed blockers for these TRP channels. These were capsazepine (CPZ), 2-aminoethyl diphenylborinate (2-APB), clotrimazole (CTZ), and 2-(12-hydroxydodeca-5,10-diynyl)-3,5,6-trimethyl-p-benzoquinone (AA861), previously shown to block recombinant TRPV1 (McIntyre et al., 2001), TRPC1 (Lievremont et al., 2005), TRPM2 (Kheradpezhouh et al., 2014), and TRPM7 (Chen et al., 2010), respectively.

**Figure 4:**
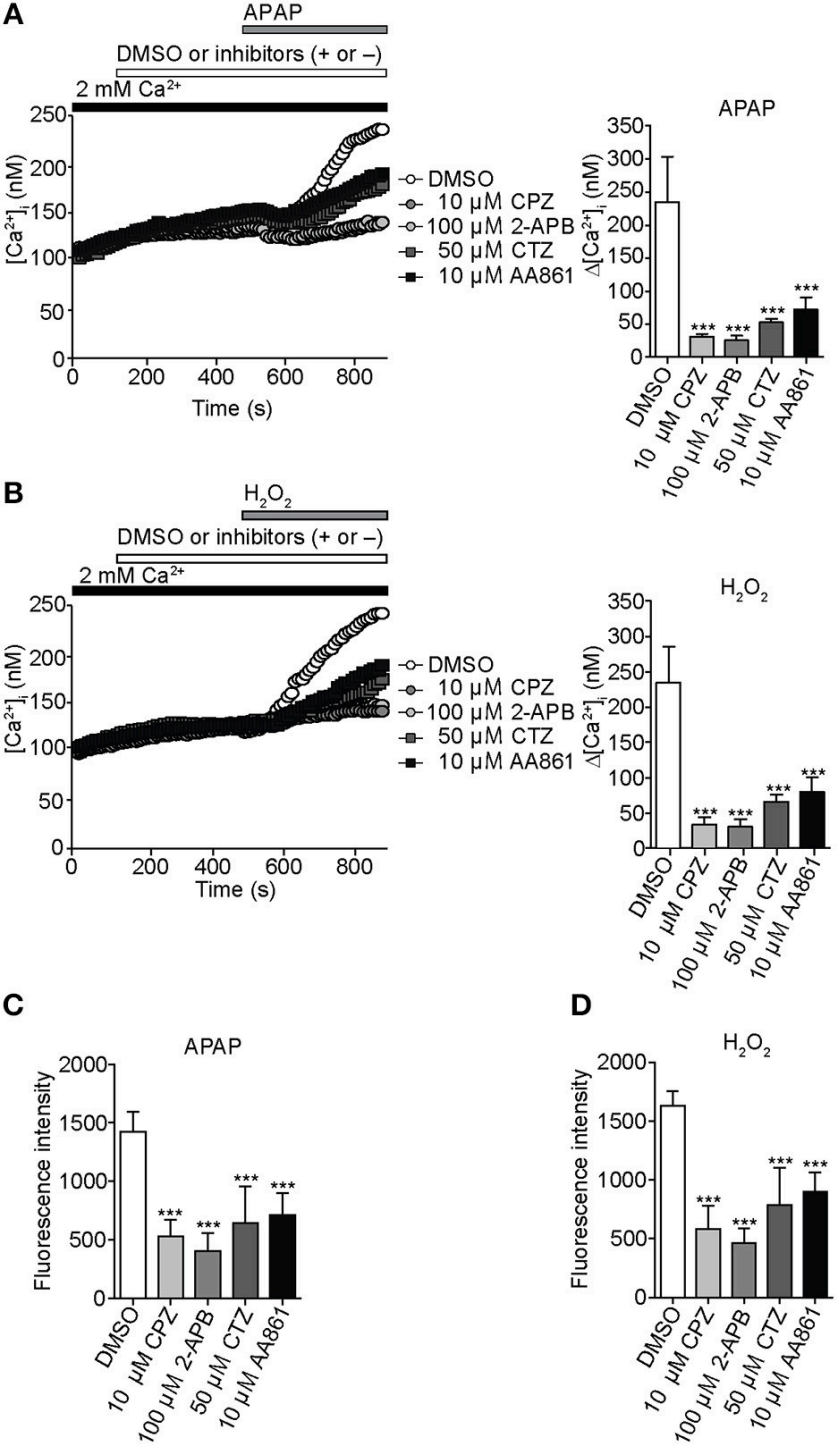
**Inhibition of APAP- or H_2_O_2_-induced Ca^2+^ entry and ROS production by selective TRP channel blockers in HepG2 cells**. **(A)** Effects of selective blockers capsazepine (CPZ; 10 μM) for TRPV1, 2-APB (100 μM) for TRPC1, clotrimazole (CTZ; 50 μM) for TRPM2, or AA861 (10 μM) for TRPM7 on [Ca^2+^]_i_ rises evoked by APAP (20 mM). Average time courses (left) and Δ[Ca^2+^]_i_ (right) (*n* = 18–57). **(B)** Effects of 10 μM CPZ, 100 μM 2-APB, 50 μM CTZ, and 10 μM AA861 on [Ca^2+^]_i_ rises evoked by H_2_O_2_ (1 mM). Average time courses (left) and Δ[Ca^2+^]_i_ (right) (*n* = 19–43). **(C,D)** Effects of selective blockers of TRP channels on ROS production evoked by APAP (20 mM) **(C)** or H_2_O_2_ (1 mM) **(D)**. Blockers are applied for 3 h before and during APAP or H_2_O_2_ stimulation. Data points are mean ± SEM. ^***^P < 0.001 compared to DMSO. All data of [Ca^2+^]_i_ measurements were analyzed by Student's *t*-test, while those of ROS measurements were analyzed by ANOVA and Bonferroni *post-hoc*.

For [Ca^2+^]_i_ measurements, HepG2 cells were pretreated with 10 μM CPZ, 100 μM 2-APB, 50 μM CTZ, or 10 μM AA861 for 6 min, then with APAP (20 mM) or H_2_O_2_ (1 mM). [Ca^2+^]_i_ increases induced by APAP in HepG2 cells were significantly lower if cells were also pretreated with CPZ, 2-APB, CTZ, or AA861 (Figure 4A). Strikingly, CPZ and 2-APB exerted relatively strong, however insignificantly inhibition among the blockers employed. Similar inhibition was observed against [Ca^2+^]_i_ increases induced by 1 mM H_2_O_2_ (Figure 4B). Levels of intracellular ROS at 3 h after incubation of HepG2 with either APAP (Figure 4C) or H_2_O_2_ (Figure 4D) were also significantly suppressed by pretreatment with the four TRP channel blockers. Treatment of HepG2 cells with TRP channel blockers alone did not affect ROS levels (Supplementary Figure 1C). Thus, native TRPV1, TRPC1, TRPM2, and TRPM7 are likely to be involved in the APAP-induced oxidative stress and Ca^2+^ overload we observed in HepG2 cells.

**Figure 5:**
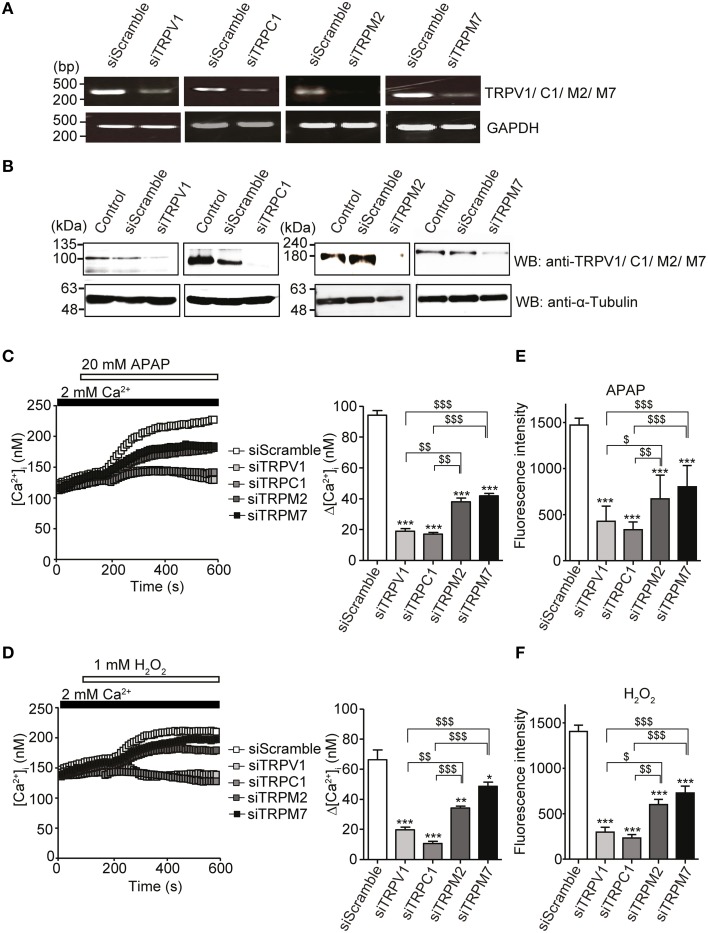
**Inhibition of APAP- or H_2_O_2_-induced Ca^2+^ responses and ROS production by siRNA-mediated knockdown of either TRPV1, TRPC1, TRPM2, or TRPM7 channel in HepG2 cells**. **(A)** Effects of specific siRNA (siTRP) on channels of TRPV1, TRPC1, TRPM2, and TRPM7 mRNA levels detected by RT-PCR. GAPDH is used as a positive control. **(B)** Effects of siTRPV1, siTRPC1, siTRPM2, and siTRPM7 on levels of corresponding proteins detected by western blot. An anti-α-tubulin is used as a loading control. **(C,D)** [Ca^2+^]_i_ changes induced by 20 mM APAP **(C)** or 1 mM H_2_O_2_ **(D)** in cells treated with siTRPM2, siTRPM7, siTRPV1, siTRPC1, or siScramble. Average time courses (left) and Δ[Ca^2+^]_i_ (right) (*n* = 50–125). **(E,F)** Inhibitory effects of siTRPM2, siTRPM7, siTRPV1, siTRPC1, or siScramble on ROS produced by 20 mM APAP **(E)** or 1 mM H_2_O_2_ for 3 h **(F)**. Data points are mean ± SEM. ^*^P < 0.05, ^**^P < 0.01 and ^***^P < 0.001 compared to siScramble. ^$^*P* < 0.05, ^$$^*P* < 0.01, and ^$$$^*P* < 0.001 compared to siTRPV1 or siTRPC1. All data of [Ca^2+^]_i_ measurements were analyzed by Student's *t*-test, while those of ROS measurements were analyzed by ANOVA and Bonferroni *post-hoc*.

To confirm this interpretation, knockdown of individual channels was accomplished by treating HepG2 cells with a small interfering RNA (siRNA) duplex targeting either TRPV1, TRPC1, TRPM2, or TRPM7. Reduced mRNA (Figure 5A) and protein levels (Figure 5B, Supplementary Figure 1D) of the corresponding TRPV1, TRPC1, TRPM2, and TRPM7 channels were observed with specific siRNA, as compared with control siRNA (siScramble). Importantly, HepG2 cells transfected with siTRPV1 or siTRPC1 showed a significant reduction in APAP- or H_2_O_2_-induced Ca^2+^ influx (Figures 5C,D, respectively) and intracellular ROS levels (Figures 5E,F, respectively). This effect was greater than that in HepG2 cells transfected with siTRPM2 or siTRPM7. No significant changes in ROS levels were found in unstimulated HepG2 cells transfected with each of these siRNAs, as compared with siScramble (Supplementary Figure 1E). These results suggest that TRPV1 and TRPC1 were more involved in APAP-induced oxidative stress and Ca^2+^ overload in HepG2 cells than were TRPM2 and TRPM7.

### Apoptosis is involved in APAP or H_2_O_2_-induced HepG2 cell death

**Figure 6:**
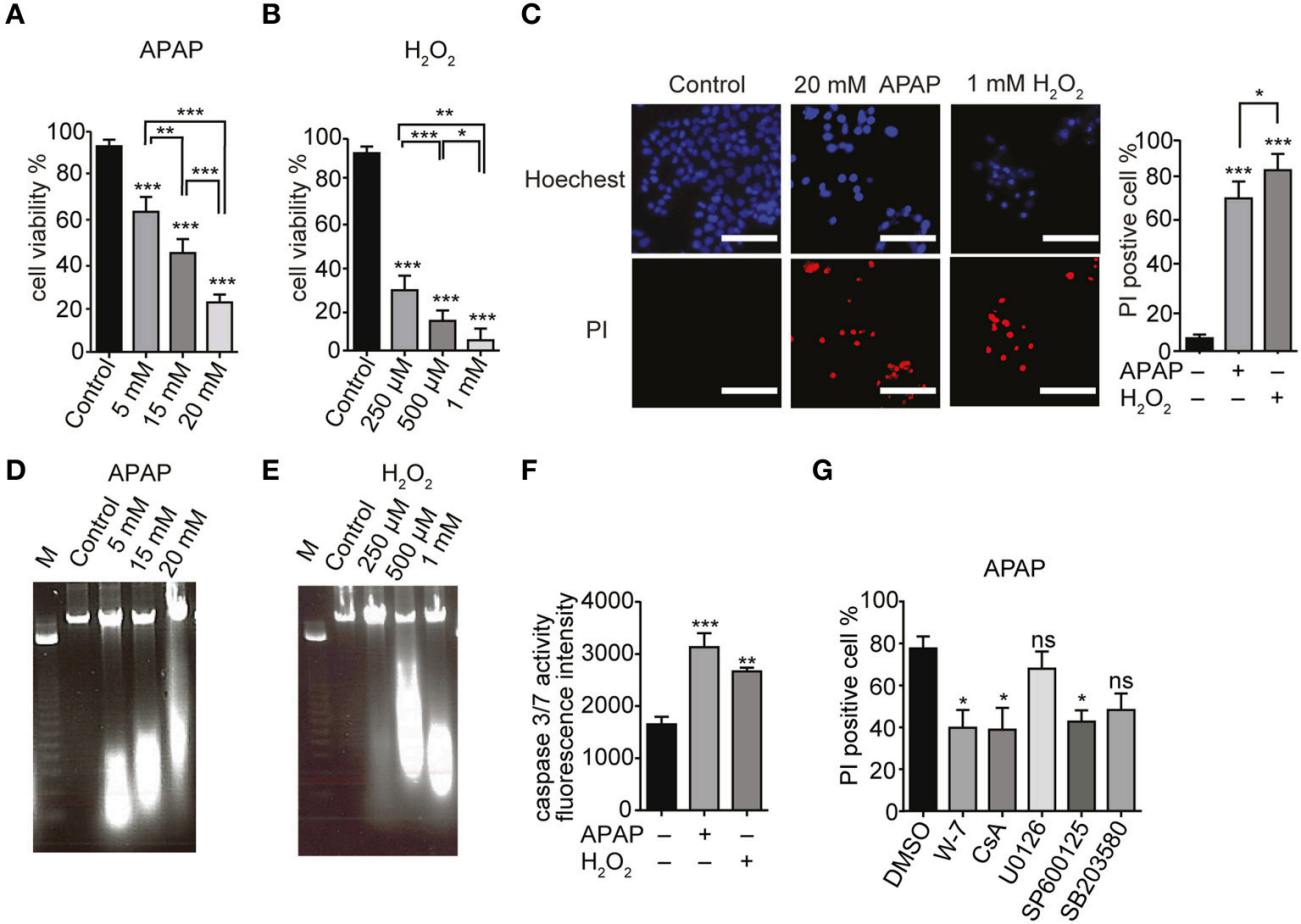
**APAP-induced HepG2 cell death**. **(A,B)** Dose dependence of viabilities of HepG2 cell treated with APAP **(A)** or H_2_O_2_ **(B)** for 24 h. Cell viability is presented as percentage of viable cells in trypan blue exclusion assay. **(C)** HepG2 cell death upon exposure to 20 mM APAP or 1 mM H_2_O_2_ for 24 h. Hoechst33342- and propidium iodide (PI)-staining were visualized by fluorescence microscopy. Scale bar, 100 μm. **(D,E)** DNA fragmentation in HepG2 cells. Cells were exposed for 24 h to APAP **(D)** (5, 15, and 20 mM) or H_2_O_2_ **(E)** (250 μM, 500 μM, and 1 mM). M is the DNA marker. **(F)** Caspase 3/7 activity in HepG2 cells stimulated with 20 mM APAP or 1 mM H_2_O_2_ for 24 h. **(G)** Effects of either Ca^2+^-calmodulin antagonist (W-7; 1 μM), cyclosporine A (CsA; 4 μM), or different MAPK-specific inhibitors (U0126, SP600125 and SB203580; 20 μM) on HepG2 cell death induced by APAP for 24 h in Hoechst33342- and PI-staining assays. Data points are mean ± SEM. *P*≥0.05, ^*^P < 0.05, ^**^P < 0.01, and ^***^P < 0.001 compared to DMSO or control. Differences not statistically significant are labeled as (ns). All data were analyzed by ANOVA and Bonferroni *post-hoc*.

Acetaminophen has been reported to induce cell death by apoptosis (Boulares et al., 2002). HepG2 cells were cultured in serum-free DMEM and treated with different doses of APAP (5, 15, and 20 mM; Figure 6A) or H_2_O_2_ (0.25, 0.5, and 1 mM; Figure 6B) for 24 h. A dose dependent decrease in HepG2 cell viability was shown by trypan blue exclusion. Similar dose dependent decreases in cell viability were also observed with 6 and 12 h treatments with APAP (Supplementary Figure 2A) or H_2_O_2_ (Supplementary Figure 2B). HepG2 cell death, assessed by Hoechst33342/PI staining, indicated a significant increase in the number of PI-positive dead cells (red) after incubation with 20 mM APAP or 1 mM H_2_O_2_ for 24 h (Figure 6C).

Internucleosomal DNA fragmentation and caspase 3/7 activation represent essential steps in the apoptotic cell death (Denault and Salvesen, 2002). HepG2 cells treated with APAP or H_2_O_2_ for 24 h showed a dose dependent increase in DNA fragmentation compared with control cells (Figures 6D,E, respectively). To examine whether HepG2 cell death elicited by APAP or H_2_O_2_ occurs *via* apoptosis, we measured the caspase 3/7 activity. There was an increase in caspase 3/7 activity in HepG2 cells proportional to the duration of treatment with either APAP (20 mM) or H_2_O_2_ (1 mM) (Figure 6F, Supplementary Figure 2C). These results suggest that APAP and H_2_O_2_ induce apoptotic death of HepG2 cells.

We next assessed involvement of ERK, JNK or P38 MAPK pathways in APAP-induced HepG2 cell death. These pathways regulate the cell cycle, differentiation and growth as well as apoptosis (Tidyman and Rauen, 2009) and the mitochondrial permeability transition (MPT; Qian et al., 1997). For these experiments, we employed the specific inhibitors W-7 for Ca^2+^-calmodulin, cyclosporine A (CsA) for MPT, U0126 for ERK, SP600125 for JNK, and SB203580 for P38 MAPK. Compared with untreated cells, there was no significant cell death in HepG2 cells treated with 1 μM W-7, 4 μM CsA, or 20 μM of either U0126, SP600125 or SB203580 (Supplementary Figure 2D). HepG2 cells treated with some of these inhibitors were partially protected from APAP compared to the cells that were treated only with APAP. However, at 20 μM, U0126 or SB203580 did not significantly decrease APAP-induced cell death (Figure 6G, Supplementary Figure 2E). This finding suggests that APAP induced apoptosis in HepG2 cells was *via* activation of JNK and MPT, while the p38 and the ERK MAPK pathways did not contribute.

**Figure 7:**
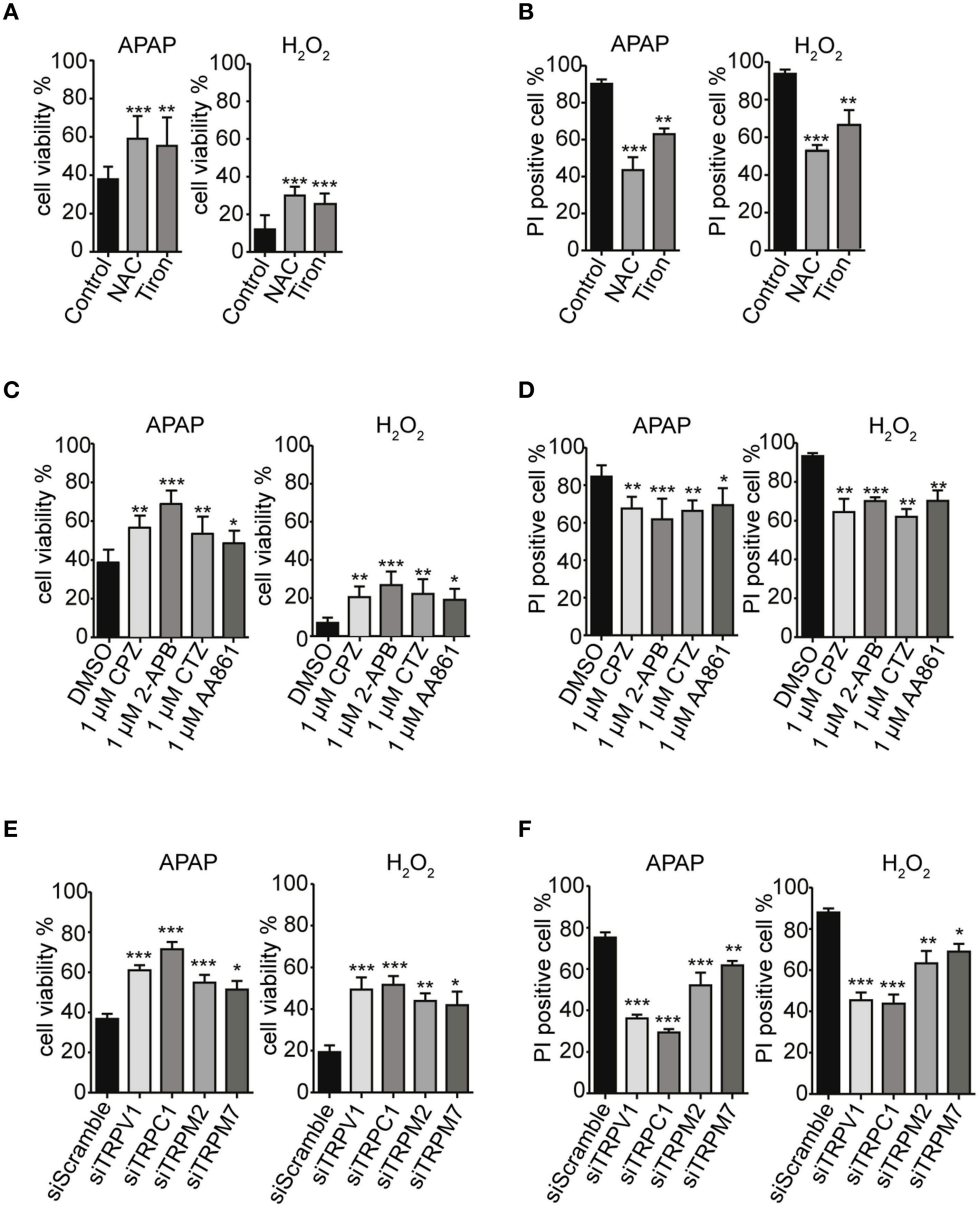
**TRPV1, TRPC1, TRPM2, and TRPM7 confer susceptibility to APAP-induced cell death ***via*** ROS in HepG2 cells**. **(A,B)** ROS scavengers, NAC and tiron (1 mM), suppressed APAP- or H_2_O_2_-induced losses of cell viability **(A)** and increases of cell death **(B)** in HepG2 cells. **(C,D)** Selective TRP channels blockers, 1 μM of either CPZ, 2-APB, CTZ, or AA861 suppressed APAP- or H_2_O_2_-induced losses of cell viability **(C)** and increases of cell death **(D)** in HepG2 cells. **(E,F)** siRNA-mediated knockdown of TRPV1, TRPC1, TRPM2, and TRPM7 suppressed APAP- or H_2_O_2_-induced losses of cell viability **(E)** and increases of cell death **(F)** in HepG2 cells. Inhibitors are applied for 3 h before and during APAP or H_2_O_2_ stimulation. Data points are mean ± SEM. ^*^P < 0.05, ^**^P < 0.01, and ^***^P < 0.001 compared to the DMSO, siScramble or control. All data were analyzed by ANOVA and Bonferroni *post-hoc*.

To investigate the correlations among ROS levels, [Ca^2+^]_i_ elevation and cell death, we examined effects of ROS scavengers on HepG2 cell viability in the presence of APAP or H_2_O_2_. Preincubation of HepG2 cells with of either NAC or tiron (1 mM) for 3 h prior to treatment with APAP or H_2_O_2_ significantly improved cell viability (Figure 7A) and reduced the number of PI-positive cells (red) (Figure 7B, Supplementary Figure 3A). The ROS scavengers alone showed no significant effects on cell viability or PI-staining in HepG2 cells (Supplementary Figures 4A,B).

Strikingly, pretreatment of HepG2 cells with TRP channel blockers (1 μM of either CPZ, 2-APB, CTZ, or AA861) for 3 h prior to treatment with APAP or H_2_O_2_ significantly improved cell viability (Figure 7C) and reduced the numbers of PI-positive cells (red) (Figure 7D, Supplementary Figure 3B). Similar results were observed after siRNA-mediated knockdown of TRPV1, TRPC1, TRPM2, and TRPM7 (Figures 7E,F, Supplementary Figure 3C). Being consistent with the contribution of TRPV1 and TRPC1 to APAP-induced Ca^2+^ entry, the siRNAs for TRPV1 and TRPC1 worked more efficiently than those of the others, a finding consistent with measurements of Ca^2+^ responses (Figures 5C,D) and ROS levels (Figures 5E,F). The channel blockers or siRNAs alone did not significantly affect cell viability and number of PI-positive cell compared to untreated HepG2 cells (Supplementary Figures 4C–F).

**Figure 8:**
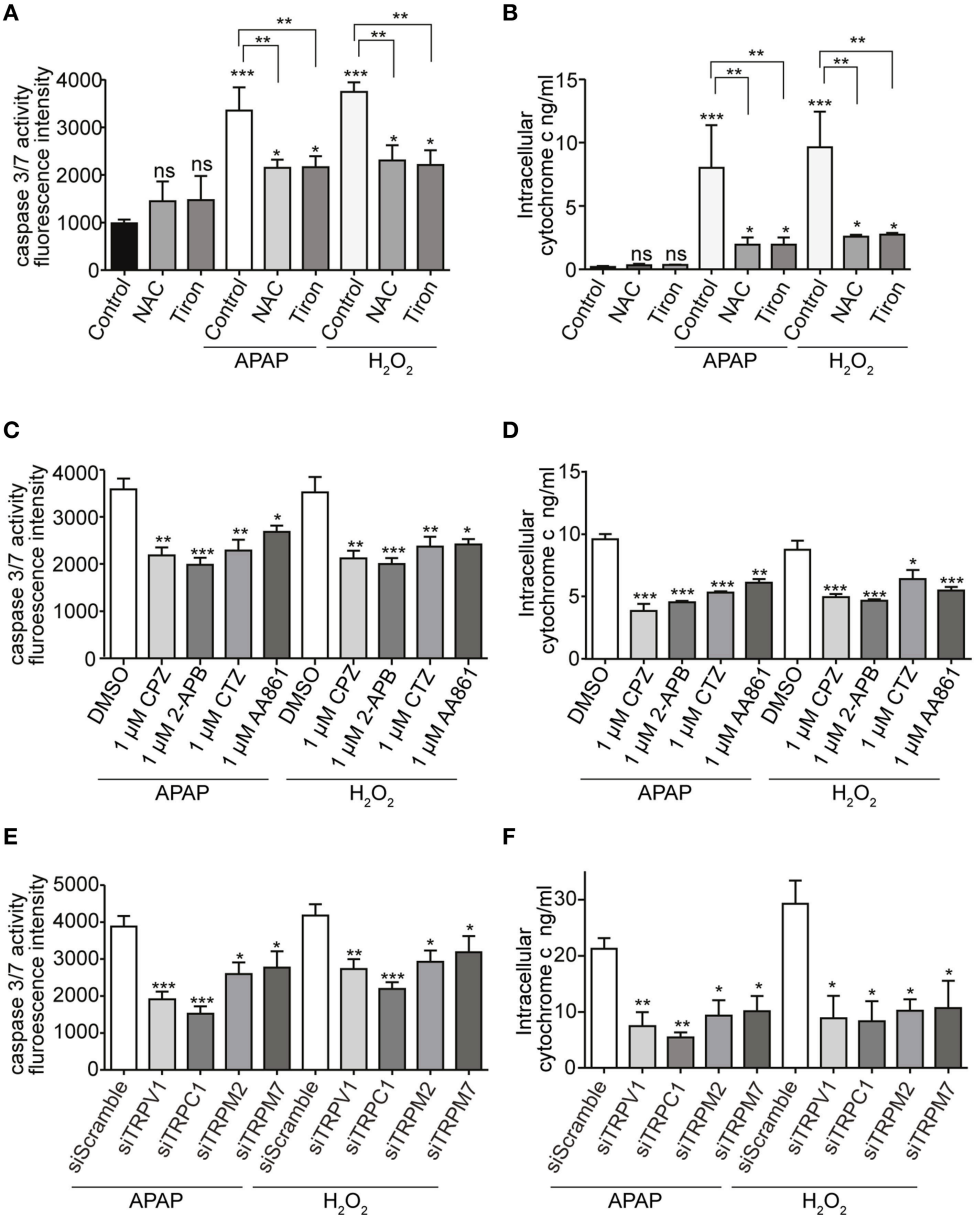
**Effects of APAP on the activity of caspase 3/7 and the level of intracellular cytochrome c in HepG2 cell**. **(A,B)** ROS scavengers, NAC or tiron, (1 mM) suppressed APAP- or H_2_O_2_-induced increases of the activity of caspase 3/7 **(A)** and the level of intracellular cytochrome c **(B)** in HepG2 cells. **(C,D)** Selective Ca^2+^ channels blockers, 1 μM of either CPZ, 2-APB, CTZ, and AA861 suppressed APAP- or H_2_O_2_-induced increases of the activity of caspase 3/7 **(C)** and the level of intracellular cytochrome c **(D)** of HepG2 cells. **(E,F)** siRNA-mediated knockdown of TRPV1, TRPC1, TRPM2, and TRPM7 suppressed APAP- or H_2_O_2_-induced increases of the activity of caspase 3/7 **(E)** and the level of intracellular cytochrome c **(F)** in HepG2 cells. Data points are mean ± SEM. *P*≥0.05, ^*^P < 0.05, ^**^P < 0.01, and ^***^P < 0.001 compared to the DMSO, siScramble or control. Differences not statistically significant are labeled as (ns). All data were analyzed by ANOVA and Bonferroni *post-hoc*.

Increases in caspase 3/7 activity and intracellular cytochrome c release, induced by APAP or H_2_O_2_, were also ameliorated by pretreatment with ROS scavengers (1 mM of either NAC or tiron; Figures 8A,B), selective TRP channel blockers (1 μM of either CPZ, 2-APB, CTZ, or AA861; Figures 8C,D) as well as by siRNA-mediated knockdown of TRPV1, TRPC1, TRPM2, or TRPM7 (Figures 8E,F; Supplementary Figures 4G–J). These results suggest that APAP-induced HepG2 cell apoptosis was more dependent on TRPV1 and TRPC1 than on TRPM2 and TRPM7, when assessed by Ca^2+^ entry, ROS levels, and mitochondrial membrane depolarization.

### Involvement of cysteine residues in redox and APAP sensing of TRPV1 and TRPC1

**Figure 9:**
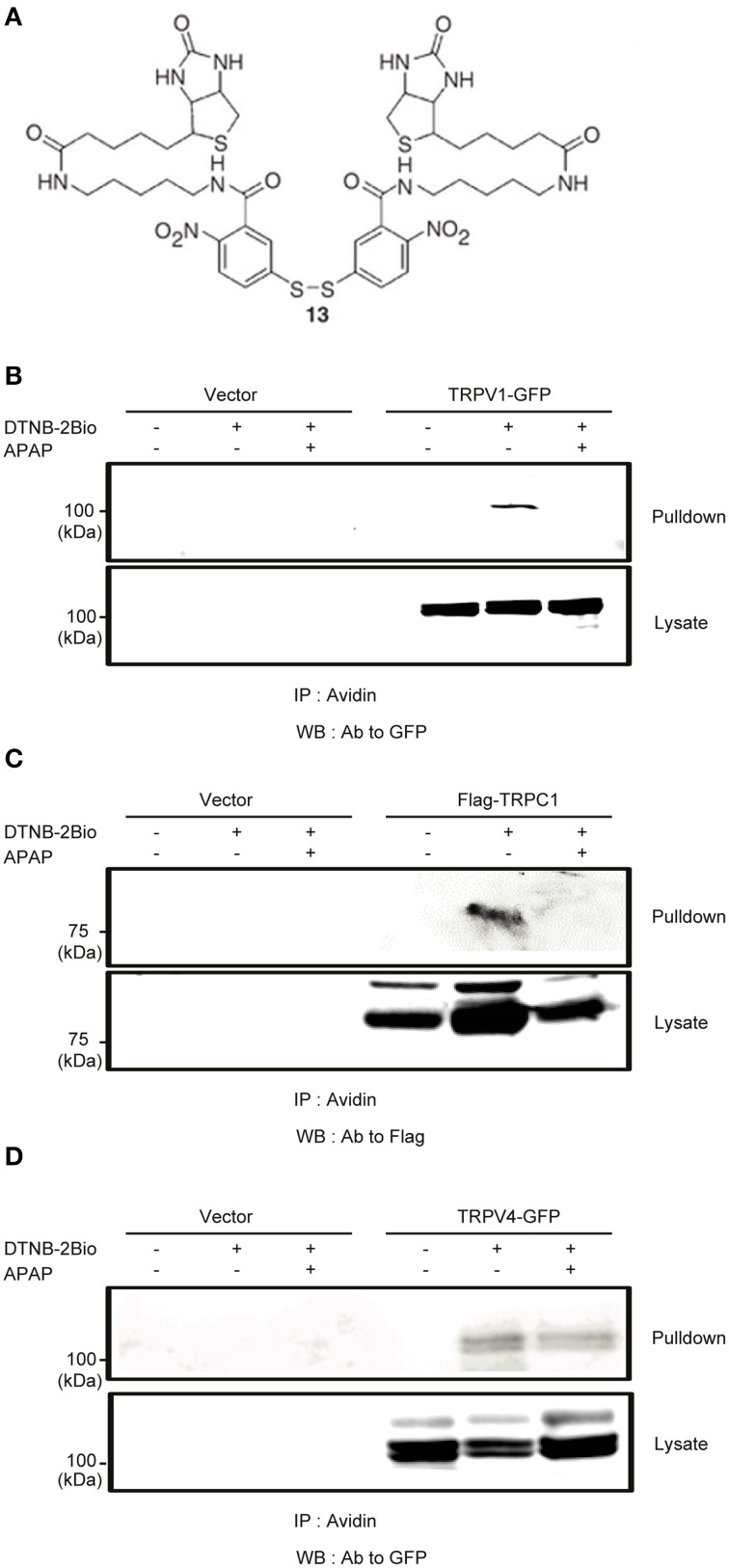
**The effects of APAP on the covalent incorporation of DTNB-2Bio in TRPV1, TRPC1, and TRPV4 proteins. (A)** Chemical structure of DTNB-2Bio (Yoshida et al., 2006). **(B–D)** Western blot using an anti-GFP or anti-Flag antibody of avidin-bound fractions (top panel) and total cell lysates (bottom panel) prepared from HEK293T cells which were transfected with EGFP-TRPV1 **(B)**, TRPC1-Flag **(C)**, EGFP-TRPV4 **(D)**, and then incubated with DTNB-2Bio in the presence or absence of 20 mM APAP.

An intriguing question is the mechanism that explains the relative importance of TRPV1 and TRPC1 compared with other TRPs. It has been reported that free thiol groups of cysteine residues are among the major targets of oxidation and electrophiles in proteins. This is also the case for TRP channels; oxidants and electrophiles induce their activation (Yoshida et al., 2006; Takahashi et al., 2008). To examine involvements of cysteine residues of TRPV1 and TRPC1 in their APAP responsiveness, we incubated permeabilized HEK293T cells transfected with GFP-tagged TRPV1 or Flag-tagged TRPC1 with DTNB-2Bio, which reacts with free thiol groups and can be purified by avidin-based affinity purification (Yoshida et al., 2006; Takahashi et al., 2008). DTNB-2Bio was incorporated into TRPV1 (Figure 9B) and TRPC1 (Figure 9C), and this was blocked by pretreatment with 20 mM APAP. In contrast, no blockade by APAP was detected for DTNB-2Bio incorporation into TRPV4, which had shown only a minor contribution to APAP-induced responses, as compared with TRPV1 and TRPC1 (Figure 9D). Thus, the action of APAP *via* the modification target cysteine residues may at least in part account for the important roles of TRPV1 or TRPC1 in APAP-induced responses in human hepatoma cells.

### GSH depletion modulates sensitivity of Ca^2+^ influx and cell death to APAP in HepG2 cells

In addition to those of ROS, glutathione (GSH) levels serve as pivotal regulators of cellular redox status. Previous studies showed that intracellular GSH suppressed oxidative stress-induced TRPM2 activation and Ca^2+^ entry in DRG neurons (Nazıroğlu et al., 2011). It is, therefore, important to study the role of GSH in suppressing APAP-induced ROS increases, Ca^2+^ overload *via* TRP channels, and cell death in HepG2 cells. Moreover, serum deprivation induces excess ROS production linked to depletion of intracellular GSH (Pandey et al., 2003). We found that serum deprivation enhanced the Ca^2+^ responses and cell death induced by 20 mM APAP or 1 mM H_2_O_2_ (Supplementary Figures 5A,B) in HepG2, as compared with that in normally cultured cells. However, serum deprivation did not affect caspase 3/7 activity (Supplementary Figure 5C), though the GSH content of serum-deprived HepG2 was significantly lower than that of cells cultured normally (Supplementary Figure 5D). Pretreatment of serum-deprived HepG2 with ROS scavengers (NAC or tiron at 1 mM) for 3 h significantly reversed APAP- or H_2_O_2_-induceddepletion of GSH content (Supplementary Figure 5E).

**Figure 10:**
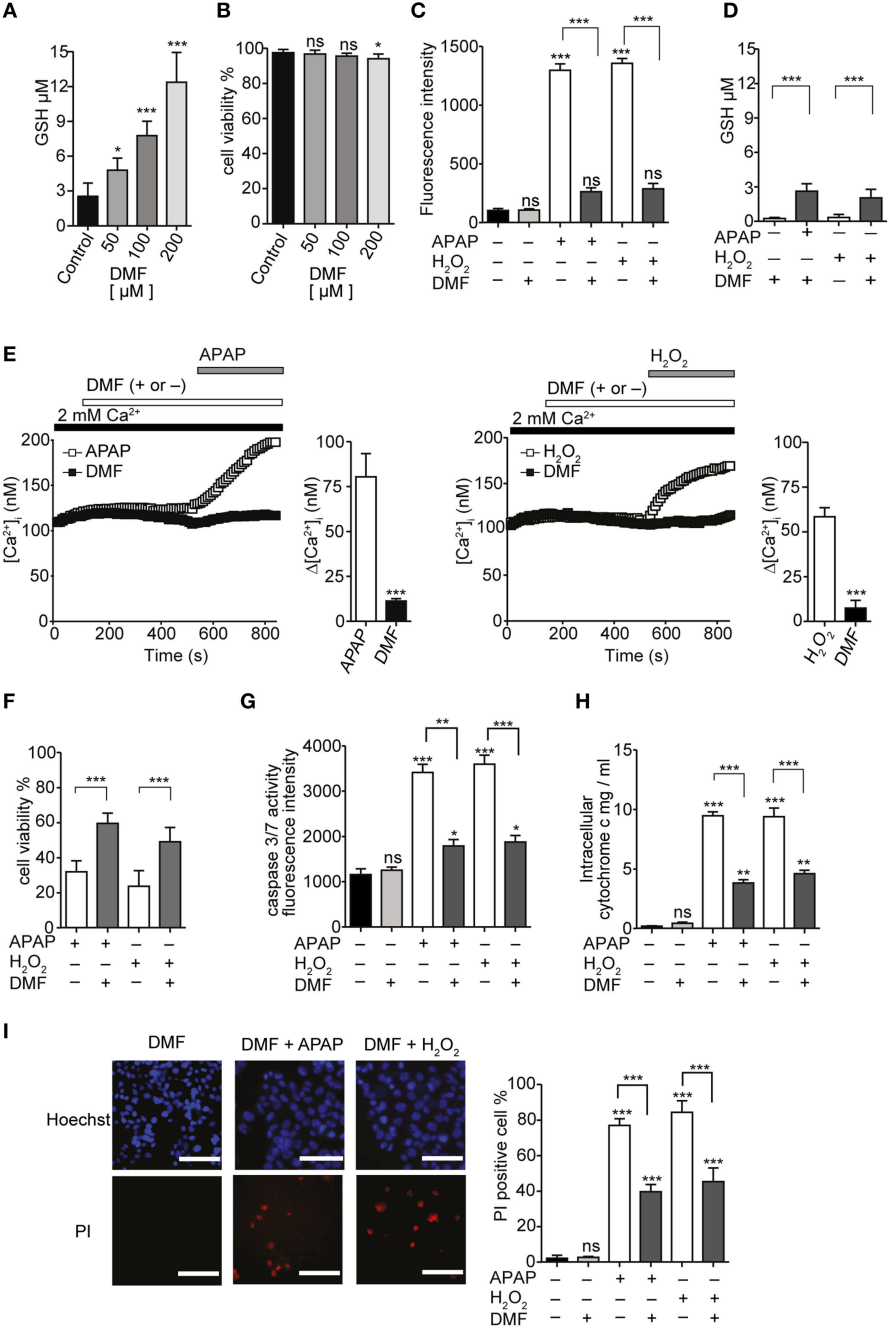
**GSH inducer DMF attenuate APAP- or H_2_O_2_-induced Ca^2+^ influx and ROS-mediated death in HepG2 cells. (A)** The effects of 50, 100, and 200 μM DMF on GSH content in HepG2 cells. **(B)** The effects of 50, 100 and 200 μM DMF on cell viabilities in HepG2 cells. **(C,D)** DMF attenuated APAP- or H_2_O_2_-induced increases of ROS levels **(C)** and GSH depletion **(D)**. **(E)** Averaged time courses and Δ [Ca^2+^]_i_ of APAP-(left) or H_2_O_2_-induced Ca^2+^ response (right) in the presence and absence of 100 μM DMF. **(F–I)** Effects of DMF on APAP- or H_2_O_2_-induced losses of cell viability **(F)** and increases of the activity of caspase 3/7 **(G)**, the level of intracellular cytochrome c **(H)**, and the percentages of PI-positive cells **(I)** in HepG2 cells. *P*≥0.05, ^*^P < 0.05, ^**^P < 0.01, and ^***^P < 0.001 compared to DMSO or control. Differences not statistically significant are labeled as (ns). Data points are mean ± SEM. All data of [Ca^2+^]_i_ measurements were analyzed by Student's *t*-test, while other data were analyzed by ANOVA and Bonferroni *post-hoc*.

Dimethylfumarate (DMF) was previously reported to upregulate antioxidant activities and suppress cell death induced by H_2_O_2_ (Duffy et al., 1998). Treatment of serum-deprived HepG2 with 50, 100, or 200 μM DMF for 24 h significantly increased GSH content in a dose-dependent manner (Figure 10A). There were no significant changes in cell viability, except for a significant decrease with 200 μM DMF (Figure 10B). Intracellular ROS levels 3 h after incubation of HepG2 with either 20 mM APAP or 1 mM H_2_O_2_ were also significantly reduced by pretreatment with 100 μM DMF (Figure 10C). Furthermore, GSH content in HepG2 cells after 24 h incubation with either APAP or H_2_O_2_ was also significantly higher in cells that had been pretreated with 100 μM DMF (Figure 10D).

Preincubation of serum-deprived HepG2 cells with 100 μM DMF for 24 h, followed by pretreatment with 100 μM DMF for 6 min, attenuated the [Ca^2+^]_i_ increases induced by 20 mM APAP or 1 mM H_2_O_2_ (Figure 10E). In addition, DMF attenuated the loss of cell viability (Figure 10F), increase in caspase 3/7 activity (Figure 10G), intracellular cytochrome c levels (Figure 10H), and cell death (Figure 10I) induced by APAP or H_2_O_2_. No significant cytotoxicity was observed when DMF was administrated in combination with APAP as shown in Figure 10. These results support a role for GSH depletion, reversible with GSH inducer DMF, in APAP-induced oxidative stress, Ca^2+^ influx, and HepG2 cell death.

### Expression and cellular localization of native redox-sensitive TRP channels in the human liver

**Figure 11:**
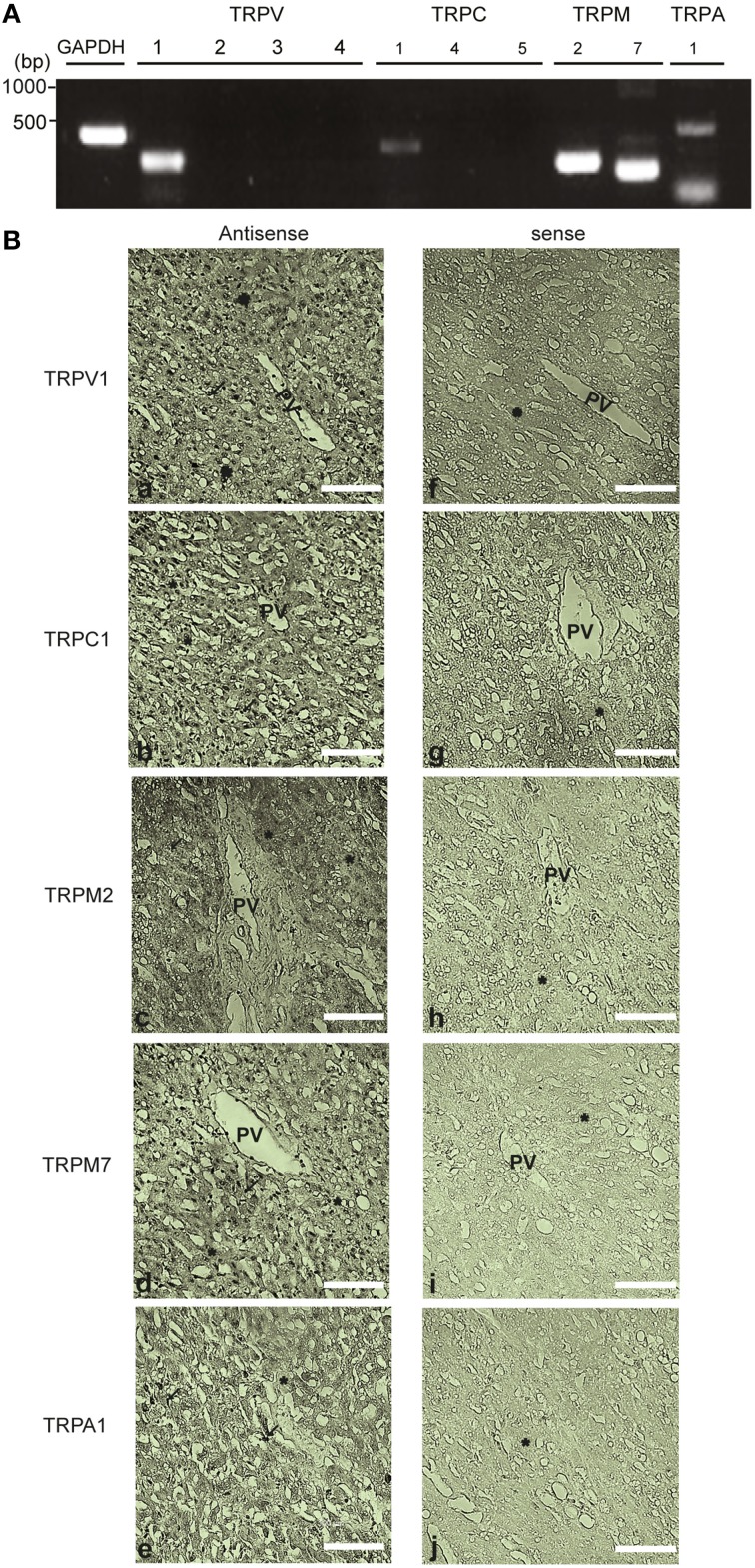
**Expression and localization of redox-sensitive TRP channel in the human liver tissue**. **(A)** Expression of mRNAs for TRPV1-4, TRPC1, TRPC4, TRPC5, TRPM2, TRPM7, TRPA1, and GAPDH detected by PCR in the cDNA library from normal human liver. Specific PCR primers used are listed in the Table 1. **(B)** *In situ* hybridization analysis of TRPV1, TRPC1, TRPM2, TRPM7, and TRPA1 mRNAs in the paraffin section of the human liver. mRNAs of TRPV1 **(a,f)**, TRPC1 **(b,g)**, TRPM2 **(c,h)**, TRPM7 **(d,i)** were localized in hepatocytes (^*^) and Kupffer cells (arrow). TRPA1 **(e,j)** was localized in the lining of sinusoids and Kupffer cells of the human liver tissue. The images from the analysis of both the antisense (left) and sense probes (right) are shown. Scale bar, 100 μm.

To confirm the validity of using HepG2 cells as a model system for hepatotoxicity, we conducted histological experiments to obtain definitive evidence that redox sensitive TRP channels are expressed in the human liver. Expression profiles of TRPV1-4, TRPC1, TRPC4, TRPC5, TRPM2, TRPM7, and TRPA1 were analyzed using PCR in cDNA of normal human liver. TRPV1, TRPC1, TRPM2, TRPM7, and TRPA1 were expressed (Figure 11A).

*In situ* hybridization of normal human liver sections with antisense probes yielded strong staining for TRPV1, TRPC1, TRPM2, and TRPM7, localized in the nuclei of hepatocytes and Kupffer cells (Figure 11B). The sinusoidal endothelial lining and Kupffer cells were also positive for TRPA1 expression but at a lower level, while hepatocytes were negative for this channel (Figure 11B). No hybridization signal was observed with sense probes applied as negative controls (Figure 11B). Recombinant TRPV1, TRPC1, TRPM2, TRPM7, and TRPA1 transcripts were intensely labeled mainly in the nucleus of HEK293T cells (Supplementary Figures 6A–E). RNA transcripts of these TRPs, except for TRPA1, were expressed in HepG2 cells (Supplementary Figures 6A–E).

## Discussion

**Figure 12:**
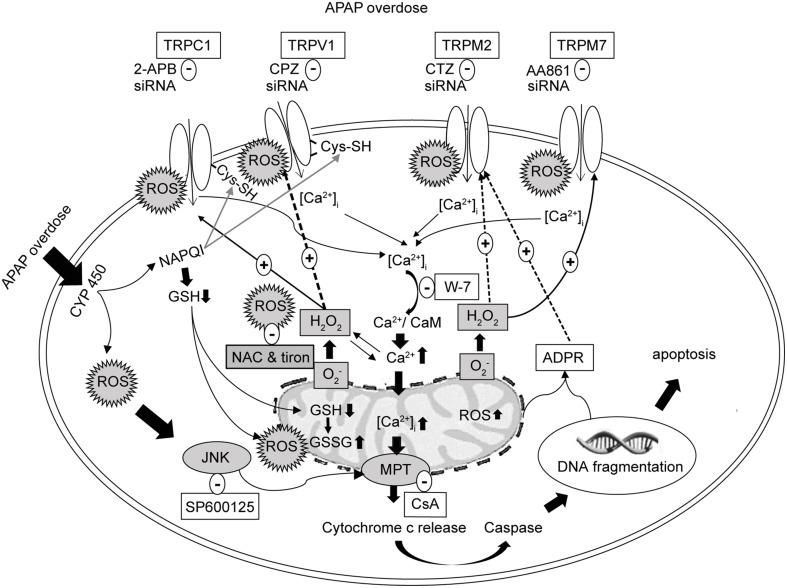
**Schematic representation of the proposed signaling mechanism underlying cell death regulated by Ca^2+^ entry via redox-sensitive TRPV1, TRPC1, TRPM2, and TRPM7 channels activated by APAP overdose in HepG2**. TRPV1 and TRPC1 channels are activated by oxidative modification of free sulfhydryl groups of cysteine residues, which APAP is able to reach to enhance activation. Initial Ca^2+^ influx by APAP-induced ROS induces the mitochondrial and nuclear damages mediating the release of ADPR, which is released into the cytosol to activate TRPM2. ROS activates TRPM7. Suppression of TRPV1, TRPC1, TRPM2, and TRPM7 using blockers, siRNAs and ROS scavengers (NAC or tiron) alleviates APAP-induced Ca^2+^ entry and HepG2 death. APAP, N-acetyl-para-aminophenol; [Ca^2+^]_i_, calcium ions; HepG2, human hepatoma cell line; TRP, transient receptor potential channels; TRPV1, type 1 vanilloid receptor; TRPC1, type 1 canonical receptor; TRPM2, type 2 melastatin receptor; TRPM7, type 7 melastatin receptor; CaMs, calmodulins; CPZ, capsazepine (TRPV1 antagonist); 2-APB, 2-aminoethyl diphenylborinate (TRPC1 antagonist); CTZ, clotrimazole (TRPM2 antagonist); AA861, 2-(12-hydroxydodeca-5,10-diynyl)-3,5,6-trimethyl-p-benzoquinone (TRPM7 antagonist); CsA, cyclosporine A (inhibitor of mitochondrial permeability transition, MPT); H_2_O_2_, hydrogen peroxide; ${\text{O}}_{2}^{-}$, superoxide anion; ROS, reactive oxygen species; GSH, glutathione; ADPR, adenosine diphosphate ribose; JNK, c-jun NH2-terminal kinase; NAC, N-acetyl-L-cysteine.

In this study, we found that APAP overdose elicited ROS production, [Ca^2+^]_i_ increases, and GSH depletion triggering HepG2 cell death. All responses were markedly reduced by pretreatment with the ROS scavengers, NAC or tiron, or with the GSH inducer DMF. In HepG2 cells, expression of redox-sensitive TRP channels including TRPV1, TRPC1, TRPM2, and TRPM7 was observed. Suppression of TRP channels, particularly, TRPV1 and TRPC1, with channel blockers and siRNAs in terms of function and expression, respectively, substantially reduced APAP-induced ROS formation, Ca^2+^ influx, and cell death, as summarized in Figure 12. Additionally, *in situ* hybridization analysis of normal human liver sections showed that transcripts for TRPV1, TRPC1, TRPM2, and TRPM7 channels were primarily localized to hepatocytes and Kupffer cells, while mRNA for TRPA1 was visible, to a lesser extent, in Kupffer cells and sinusoidal endothelium but was absent in hepatocytes.

Earlier reports described the presence of TRPV1-4, TRPC1, and TRPM7 mRNAs but not TRPC4 and TRPC5 in HepG2 cells (Vriens et al., 2004; El Boustany et al., 2008). Other studies showed expression of the TRPM7 channel in rat HSC-T6 hepatic stellate, RLC-18 and WIF-B hepatoma cells (Lam et al., 2012; Liu et al., 2012) and of the TRPC1 channel in rat H4-IIE hepatoma cells (Chen and Barritt, 2003). In addition, our study newly reveals, by RT-PCR and *in situ* hybridization, the existence of TRPM2 mRNA in HepG2 cells. Furthermore, this is the first *in situ* hybridization analysis to show localization and distribution of TRPV1, TRPC1, TRPM2, and TRPM7 transcripts in the hepatocyte of human liver. Regarding functional expression, HEK293 cells heterologously expressing TRPV1, TRPC1, TRPM2, or TRPM7 responded well to H_2_O_2_, in agreement with previous reports (Hara et al., 2002; Aarts et al., 2003; Yoshida et al., 2006; Salazar et al., 2008), and as well to APAP as indicated by increased Ca^2+^ entry. In contrast, HEK293 cells expressing TRPV2, TRPV3, TRPV4, TRPC4, or TRPC5 failed to respond to APAP. Such differences are presumably due to structural variations among various TRP channel subfamily members (Figure 3). Interestingly, it has been reported that TRPV subunits can assemble into hetero-oligomeric channel complexes, for example, between TRPV1 and TRPV2 (Hellwig et al., 2005) or TRPV1 and TRPV3 (Smith et al., 2002). Therefore, we cannot exclude the possibility that TRPV1 could operate as hetero-multimeric channel as well as homo-multimeric channel in response to APAP or H_2_O_2_.

ROS overproduction and the resulting cell damaging events are often accompanied by rises in [Ca^2+^]_i_ (Yan et al., 2006). A previous report proposed that H_2_O_2_ alters cellular redox status through increased [Ca^2+^]_i_, decreases GSH level and induces lipid peroxidation, eventually causing cell death in the human hepatoma cell line SMMC-7721 (Li et al., 2000). Other reports suggested that APAP exposure leads to increased levels of superoxide anions, which upon dismutation generate H_2_O_2_ (Dai and Cederbaum, 1995). To clarify whether such mechanisms were involved in our experiments, we used NAC, a sulfhydryl drug, which contributes to repletion of GSH by diminishing ROS production via removal of superoxide radicals, H_2_O_2_, and hydroxyl radicals (Sen and Packer, 2000). NAC is the most widely used antidote for APAP-induced hepatotoxicity (Smilkstein et al., 1991). We also employed tiron, an intracellularly active scavenger of superoxide anions and hydroxyl radicals (Krishna et al., 1992; Taiwo, 2008). There had been no previous investigations addressing whether NAC or tiron actually suppress ROS and [Ca^2+^]_i_ in HepG2 cells. Importantly, we found that pretreatments with either NAC or tiron strongly attenuated APAP-induced elevations of ROS and [Ca^2+^]_i_ in HepG2 cells. This supports the hypothesized link between APAP overdose and ROS formation in HepG2 cells. Interestingly, it was reported that NAC reduced oxidative stress and suppressed TRPV1 channel-mediated Ca^2+^ entry in neutrophils from patients with polycystic ovary syndrome (Köse and Nazıroğlu, 2015). NAC may also play a protective role against Ca^2+^ influx through regulation of TRPM2 channels in neurons (Özgül and Nazıroğlu, 2012). It was further suggested that tiron reduced Ca^2+^ entry through TRPC1 channels in mouse muscle fibers (Gervásio et al., 2008). Taken together, NAC or tiron might work through suppression of APAP-elicited oxidative stress and ensuing activation of redox-sensitive TRP channels whose molecular expression was detected in HepG2 cells.

Consistent with our results, it was reported that TRPV1, TRPC1, TRPM2, and TRPM7 channels were modulated by ROS and induced deleterious responses such as cell death (Miller, 2006; Kozai et al., 2014b). For example, TRPV1 activation has previously been shown to increase [Ca^2+^]_i_, oxidative stress and apoptotic cell death (Shin et al., 2003; Kim et al., 2006). TRPV1 and TRPC1 are activated by oxidative modification of free sulfhydryl groups of cysteine residues (Yoshida et al., 2006). We reported that TRPM2 induced cell death during conditions of oxidative stress (Hara et al., 2002). Suppression of TRPM7 expression by RNA interference blocked TRPM7 currents, anoxic Ca^2+^ influx, and ROS production, protecting cortical neurons from anoxia. This suggested a role for endogenous TRPM7 in anoxic neuronal death (Aarts et al., 2003). Thus, redox-sensitive TRP channels are widely involved in oxidative stress linked to cellular disorders.

Ca^2+^ dysregulation was reported to be involved in APAP-induced hepatocellular damage (Shen et al., 1991). It was suggested that [Ca^2+^]_i_ increases are primarily caused by inhibition of Ca^2+^-Mg^2+^ ATPase and accompany, but do not cause, hepatocellular damage (Tsokos-Kuhn et al., 1988). In our study, pretreatment with antagonists of TRPV1, TRPC1, TRPM2, and TRPM7 channels (CPZ, 2-APB, CTZ, and AA861, respectively) protected the HepG2 cells from deleterious increases in Ca^2+^ entry and ROS production and the resulting cell death induced by APAP. Importantly, knockdown of these channels by a siRNA strategy resulted in significantly suppressed Ca^2+^ overload and ROS production in response to either APAP or H_2_O_2_. CTZ was previously reported to protect the liver against normothermic ischemia-reperfusion injury in rats (Iannelli et al., 2009). Likewise, CPZ treatment conferred significant protection against liver dysfunction, as indicated by serum alanine transaminase (ALT) and aspartate transaminase (AST) activities, in sepsis (Ang et al., 2011). Moreover, 2-APB reduced APAP-induced liver injury in mouse primary hepatocytes *in vivo* (Du et al., 2013). In a recent study, AA861 pretreatment significantly attenuated acute liver failure in rats by inhibiting macrophage activation (Li et al., 2014). Hence, abundant evidence indicates that activation of these TRP channels contributes to APAP-induced hepatotoxicity, raising a possibility that their blockers can ameliorate the APAP-induced damages of human hepatocytes.

Our data demonstrate that TRPC1 and TRPV1 play prominent roles in regulation of the APAP-induced responses among TRP channels expressed in HepG2 cells. Previously, we have reported that TRPV1 and TRPC1 are directly activated through oxidative modifications of cysteine residues by ROS, RNS, and electrophiles (Yoshida et al., 2006; Takahashi et al., 2011; Kozai et al., 2014b). In addition, previous reports have shown that endogenous expression of cytochrome P450 enzymes (CYPs), which convert APAP to its oxidized metabolite N-acetyl-p-benzoquinoneimine NAPQI, were detected in HepG2 cells (Sumida et al., 2000; Boess et al., 2003; Wilkening and Bader, 2003; Maruyama et al., 2007; El Gendy and El-Kadi, 2009; Hart et al., 2010; Wang et al., 2011). It is therefore possible that TRPV1 and TRPC1 are activated by NAPQI together with ROS/RNS generated upon APAP treatment to evoke pronounced downstream responses, compared with TRPM2 and TRPM7, which is most likely to be only activated by indirect action of ROS/RNS generated upon APAP treatment (Hara et al., 2002; Aarts et al., 2003). Interestingly, TRPA1, which is the most sensitive to oxidants and electrophiles among TRP channels (Takahashi et al., 2011), has been reported to respond to NAPQI to mediate spinal nociception induced by APAP in sensory neurons (Andersson et al., 2011). Alternatively, APAP itself may act directly but non-covalently to the DTNB-2Bio target cysteine residue(s), and activates TRPV1 and TRPC1. This possibility is likely when we consider our finding that APAP efficiently activates TRPV1 and TRPC1 expressed recombinant in HEK cells, in which expression of CYPs has not been clarified yet. At this point, we cannot conclude whether the action of APAP is through covalent binding of NAPQI or non-covalent binding of APAP itself, because both possibilities are consistent with our finding that treatment of permeabilized cells with APAP suppresses incorporation of DTNB-2Bio into TRPV1 and TRPC1 proteins (Figure 9). Thus, cysteine residues susceptible to oxidative modifications are critical for exacerbation of APAP-mediated hepatotoxicity in human, suggesting that TRP channels such as TRPC1 and TRPV1 susceptible to this type of modification are potential targets for the treatment of APAP-induced liver toxicity.

In our study, decrease in HepG2 cell viability as well as increases in DNA fragmentation, intracellular cytochrome c level, and caspase 3/7 activity were observed after either APAP overdose or H_2_O_2_ treatment. These responses were significantly reversed by ROS scavengers or TRP channel blockers. APAP overdose was previously reported to cause DNA fragmentation in primary cultured mouse hepatocytes (Shen et al., 1992) and mitochondrial cytochrome c release in isolated mouse liver cells (El-Hassan et al., 2003). Kim and colleagues suggested that oxidative stress and increased Ca^2+^ levels are both stimuli of mitochondrial dysfunction in rat hepatocytes (Kim et al., 2003). Downstream signaling of Ca^2+^ influx and Ca^2+^ overload in mitochondria can cause opening of the MPT pore (Zoratti and Szabò, 1995), and eventually promote apoptosis (Liu et al., 1996; Polster and Fiskum, 2004). The time required for this signaling cascade can possibly account for the time elapsed between Ca^2+^ concentration changes that took place within minutes and the observation of maximal HepG2 cell death at 24 h or more (Zhivotovsky and Orrenius, 2011). Collectively, these results suggest that mitochondrial Ca^2+^ overload might have contributed to APAP-induced HepG2 cell death in our study. Some reports claim that caspase activation and apoptosis were suggested to only occur in HepG2 but not in primary hepatocytes (Shen et al., 1992). However, activation of caspases in APAP-induced liver injury has been reported previously (Hu and Colletti, 2010; Sharma et al., 2011; Li et al., 2013), and such activation of caspases due to APAP treatment was significantly reduced in cells treated with anti-apoptotic caspases inhibitors (Ferret et al., 2001), which altogether would support an apoptotic pathway for APAP toxicity in hepatocytes.

We demonstrated that serum-deprived HepG2 cells are more susceptible than normally cultured cells to APAP- or H_2_O_2_-induced cell damage, depletion in GSH stores, increased Ca^2+^ influx, and loss of cell viability. Serum, a mixture of hundreds of proteins, contains various factors needed for proliferation of cells in culture (van der Valk et al., 2004). Serum deprivation may induce oxidative stress, caused by excess production of ROS or decreased GSH, and this can be inhibited by intracellular antioxidants (Pandey et al., 2003; Zhuge and Cederbaum, 2006).

Intracellular GSH is important for detoxification of a variety of chemicals, acting as a reductant in the metabolism of peroxides and free radicals (Circu and Aw, 2012). In addition, GSH is a crucial endogenous agent to restore the normal cellular redox state when it is compromised. Previous studies suggested that depletion of GSH by H_2_O_2_ is important for apoptosis and that H_2_O_2_-mediated lipid peroxidation might be a primary event leading to other biochemical changes (Li et al., 2000). It is possible that a decline in GSH content during APAP treatment similarly altered the cellular redox state in HepG2 cells. Our results showed that, in HepG2 cells, the GSH inducer DMF attenuated APAP- or H_2_O_2_-induced decrease in GSH content, increased ROS levels, Ca^2+^ overload, and cell death associated events such as intracellular cytochrome c release and caspase 3/7 activation. DMF has been used successfully to treat multiple sclerosis (Fox et al., 2012), and psoriasis (Bovenschen et al., 2010), to attenuate renal fibrosis (Oh et al., 2012), and for cardioprotection (Ashrafian et al., 2012). It is believed to exert such benefits by restoring the balance between pro-oxidant and antioxidant systems during oxidative stress. DMF may therefore represent an alternative therapy to NAC, the current treatment for APAP hepatotoxicity.

In conclusion, this is the first study to systematically and comparatively analyze the contributions of redox-sensitive TRP channels such as TRPV1, TRPC1, TRPM2, and TRPM7 to human hepatoma cell death induced by APAP overdose. Our findings add considerably to current understanding of the mechanism of APAP-induced liver toxicity. Further comparative studies using various TRP knockout mice are important to establish individual roles *in vivo* of these TRP channels. Once our observation is confirmed in other models *in vitro* and *in vivo*, these TRP channels could represent potential drug targets for treating APAP overdose over a wide range of post-ingestion time.

## Authors contributions

YM, HB, and TN participated in research design; HB, DK, and TN conducted experiments and data collection; YM, HB, and TN performed data analysis and prepared figures; and YM, HB, TN, and RS wrote or contributed to the writing of the manuscript.

## Funding

This work was supported by a Grant-in-Aid for Scientific Research on Innovative Areas “Oxygen biology: a new criterion for integrated understanding of life” (No. 26111004) of The Ministry of Education, Culture, Sports, Science and Technology, Japan.

### Conflict of interest statement

The authors declare that the research was conducted in the absence of any commercial or financial relationships that could be construed as a potential conflict of interest.

## Abbreviations

2-APB: 2-aminoethyl diphenylborinate
AA861: 2-(12-hydroxydodeca-5, 10-diynyl)-3,5,6-trimethyl-p-benzoquinone
AITC: allyl isothiocyanate
APAP: N-acetyl-para-aminophenol
bp: base pair
[Ca^2+^]_i_: intracellular Ca^2+^ concentration
CPZ: capsazepine
CsA: cyclosporine A
CTZ: clotrimazole
DCF-DA: 2,7-dichlorofluorescein diacetate
DMF: dimethylfumarate
H_2_O_2_: hydrogen peroxide
HepG2: human hepatoma cell line
MAPK: mitogen activated protein kinase
MPT: mitochondrial permeability transition
NAC: N-acetyl-L-cysteine
PI: propidium iodide
RT-PCR: reverse transcriptase polymerase chain reaction
siRNA: small interfering RNA
tiron: 4,5-dihydroxy-1,3-benzene disulfonic acid disodium salt monohydrate
TRP: transient receptor potential
W-7: N-(6-aminohexyl)-5-chloro-1-naphthalene sulfonamide
WI-38: human lung fibroblast cell line.
