# Supplementary material for: Different Contribution of Redox-Sensitive Transient Receptor Potential Channels to Acetaminophen-Induced Death of Human Hepatoma Cell Line
Source: Front Pharmacol. 2016 Feb 9;7:19. doi: 10.3389/fphar.2016.00019 (PMC4746322; doi:10.3389/fphar.2016.00019)
Supplement: Supplementary file 1 [file DataSheet1.DOCX]

Supplementary Material

Different contribution of redox-sensitive transient receptor potential channels to acetaminophen-induced death of human hepatoma cell line

**Heba Badr^1^, Daisuke Kozai^1^, Reiko Sakaguchi^1,2^, Tomohiro Numata^1,3^, Yasuo Mori^1,2,3*^**

^1^Laboratory of Molecular Biology, Department of Synthetic Chemistry and Biological Chemistry, Graduate School of Engineering, Kyoto University, Kyoto, Japan

^2^World Premier International Research Initiative-Institute for Integrated Cell-Material Sciences, Kyoto University, Kyoto, Japan

^3^Laboratory of Environmental Systems Biology, Department of Technology and Ecology, Hall of Global Environmental Studies, Kyoto University, Kyoto, Japan

* Correspondence: Yasuo Mori, Laboratory of Molecular Biology, Department of Synthetic Chemistry and Biological Chemistry, Graduate School of Engineering, Kyoto University, Katsura Campus, Nishikyo-ku, Kyoto 615-8510, Japan.

[***mori@sbchem.kyoto-u.ac.jp***](mailto:mori@sbchem.kyoto-u.ac.jp)

SUPPLEMENTARY FIGURES

**Supplementary Figure 1. (A)** RT-PCR analysis of TRPA1 mRNA expression in HepG2 and WI38 cells as a positive control. **(B)** [Ca^2+^]_i_ induced by 100 µM allyl isothiocyanate (AITC) in HepG2 cells for 10 min. Average time courses (left) and Δ [Ca^2+^]_i_ (right) (n = 72-92). **(C)** Inconsiderable changes in the ROS levels in HepG2 cells treated with TRP channels blockers (CPZ, 2-APB, CTZ, and AA861; 1 µM). **(D)** Quantification of intensity of bands of TRPV1, TRPC1, TRPM2, and TRPM7 (for 2-3 independent measurements for each channel), normalized by α-tubulin is represented by percentage (%). Data points are mean ± SEM. P ≥ 0.05, ***P*<0.01, and ****P* < 0.001 compared to control or siScramble. **(E)** Inconsiderable changes in the ROS levels in HepG2 treated by siRNA-mediated knockdown of TRPV1, TR PC1, TRPM2, and TRPM7. Data points are mean ± SEM. *P* ≥ 0.05 compared to control, DMSO, or siScramble. Differences not statistically significant are labelled as (ns). All data of [Ca^2+^]_i_ measurements were analyzed by Student's t-test, while other data were analyzed by ANOVA and Bonferroni post-hoc.

**Supplementary Figure 2. APAP-induced HepG2 cell death.** **(A,B)** Dose dependence of viabilities of HepG2 cell treated with APAP **(A)** or H_2_O_2_ **(B)** for 6 and 12 h. Cell viability is presented as a percentage of viable cells in trypan blue exclusion assay. **(C)** Caspase 3/7 activity in HepG2 cells stimulated with 20 mM APAP (left) or 1 mM H_2_O_2_ (right) for 4, 6, and 12 h. **(D)** Representative images of HepG2 treated with Ca^2+^-calmodulin antagonist (W-7; 1 µM), cyclosporine A (CsA; 4 µM) or different MAPK-specific inhibitors (U0126, SP600125 and SB203580; 20 µM) for 24 h. Hoechst33342- and PI-staining were visualized by fluorescence microscopy. Scale bar, 100 µm. **(E)** Representative images of HepG2 treated with 1 µM W-7, 4 µM CsA, or MAPK inhibitors on APAP-induced cell death for 24 h. Hoechst33342- and PI-staining were visualized by fluorescence microscopy. Scale bar, 100 µm. Data points are mean ± SEM. *P* ≥ 0.05, **P* < 0.05, ***P* < 0.01, and ****P* < 0.001 compared to DMSO or control. Differences not statistically significant are labelled as (ns). All data were analyzed by ANOVA and Bonferroni post-hoc.

**Supplementary Figure 3.** **Hoechst33342- and PI-assay of HepG2 cell death.** **(A)** Representative images of HepG2 that were treated with ROS scavengers, NAC or tiron (1 mM), on 20 mM APAP- or 1 mM H_2_O_2_-induced cell death for 24 h. Hoechst33342- and PI-staining were visualized by fluorescence microscopy. Scale bar, 100 µm. **(B)** Representative images of HepG2 treated with TRP channel blockers (CPZ, 2-APB, CTZ, and AA861; 1 µM) on 20 mM APAP- or 1 mM H_2_O_2_-induced cell death for 24 h. Hoechst33342- and PI-staining were visualized by fluorescence microscopy. Scale bar, 100 µm. **(C)** Representative images of HepG2 treated with siRNA for knockdown of TRPV1, TRPC1, TRPM2, and TRPM7 on 20 mM APAP- or 1 mM H_2_O_2_-induced cell death for 24 h. Hoechst33342- and PI-staining were visualized by fluorescence microscopy. Scale bar, 100 µm.

**Supplementary Figure 4. (A,B)** Percentages of viable cells **(A)** and percentages of PI-positive cells **(B)** in HepG2 treated with NAC or tiron (1mM), for 24 h. **(C,D)** Percentages of viable cells **(C)** and percentages of PI-positive cells **(D)** in HepG2 cells treated with TRP channels blockers (CPZ, 2-APB, CTZ, and AA861; 1 µM). (E and F) Percentages of viable cells **(E)** and percentages of PI-positive cells **(F)** in HepG2 treated with siRNA for knockdown of TRPV1, TRPC1, TRPM2, and TRPM7. **(G,H)** Caspase 3/7 activity **(G)** and the level of intracellular cytochrome c **(H)** in HepG2 cell treated with TRP blockers (CPZ, 2-APB, CTZ, and AA861; 1 µM). **(I,J)** Caspase 3/7 activity **(I)** and the level of intracellular cytochrome c **(J)** in HepG2 cell treated with siRNA for knockdown of TRPV1, TRPC1, TRPM2, and TRPM7. Data points are mean ± SEM. *P* ≥ 0.05 compared to DMSO or siScramble. Differences not statistically significant are labelled as (ns). All data were analyzed by ANOVA and Bonferroni post-hoc.

**Supplementary Figure 5.** **Serum deprivation enhanced Ca^2+^ responses and death induced by APAP in HepG2 cells. (A)** [Ca^2+^]_i_ rises induced by APAP (20 mM) (left) or H_2_O_2_ (1 mM) (right) in HepG2 cells cultured in normal condition and serum-deprived HepG2 cells for 10 min. Average time courses (left) and Δ[Ca^2+^]_i_ (right) (n = 42-98). **(B)** Cell death in HepG2 cells cultured in normal condition and serum-deprived HepG2 cells upon exposure to APAP (20 mM) or H_2_O_2_ (1 mM) for 24 h. Hoechst33342- and PI-staining were visualized by fluorescence microscopy. Scale bar, 100 µm. **(C,D)** Caspase 3/7 activity **(C)** and GSH content **(D)** in HepG2 cells cultured in normal condition and serum-deprived HepG2 cells for 24 h. **(E)** ROS scavengers, NAC and tiron, (1 mM) suppressed APAP- or H_2_O_2_-induced depletion of GSH content in HepG2 cells. Data points are mean ± SEM. *P* ≥ 0.05, **P* < 0.05, ****P* < 0.001. Differences not statistically significant are labelled as (ns). All data of [Ca^2+^]_i_ measurements were analyzed by Student's t-test while other data were analyzed by ANOVA and Bonferroni post-hoc.

**Supplementary Figure 6.** ***In situ* hybridization analysis of redox-sensitive TRP channels expressed in HEK 293T and HepG2 cells.** Expression of mRNAs encoding TRPV1 **(panel A)**, TRPC1 **(panel B)**, TRPM2 **(panel C)**, TRPM7 **(panel D)**, and TRPA1 **(panel E)** are observed except for TRPA1 in HepG2 cells. The images from the analysis of both antisense (left) and sense probes (right) are shown. Scale bar, 100 µm.


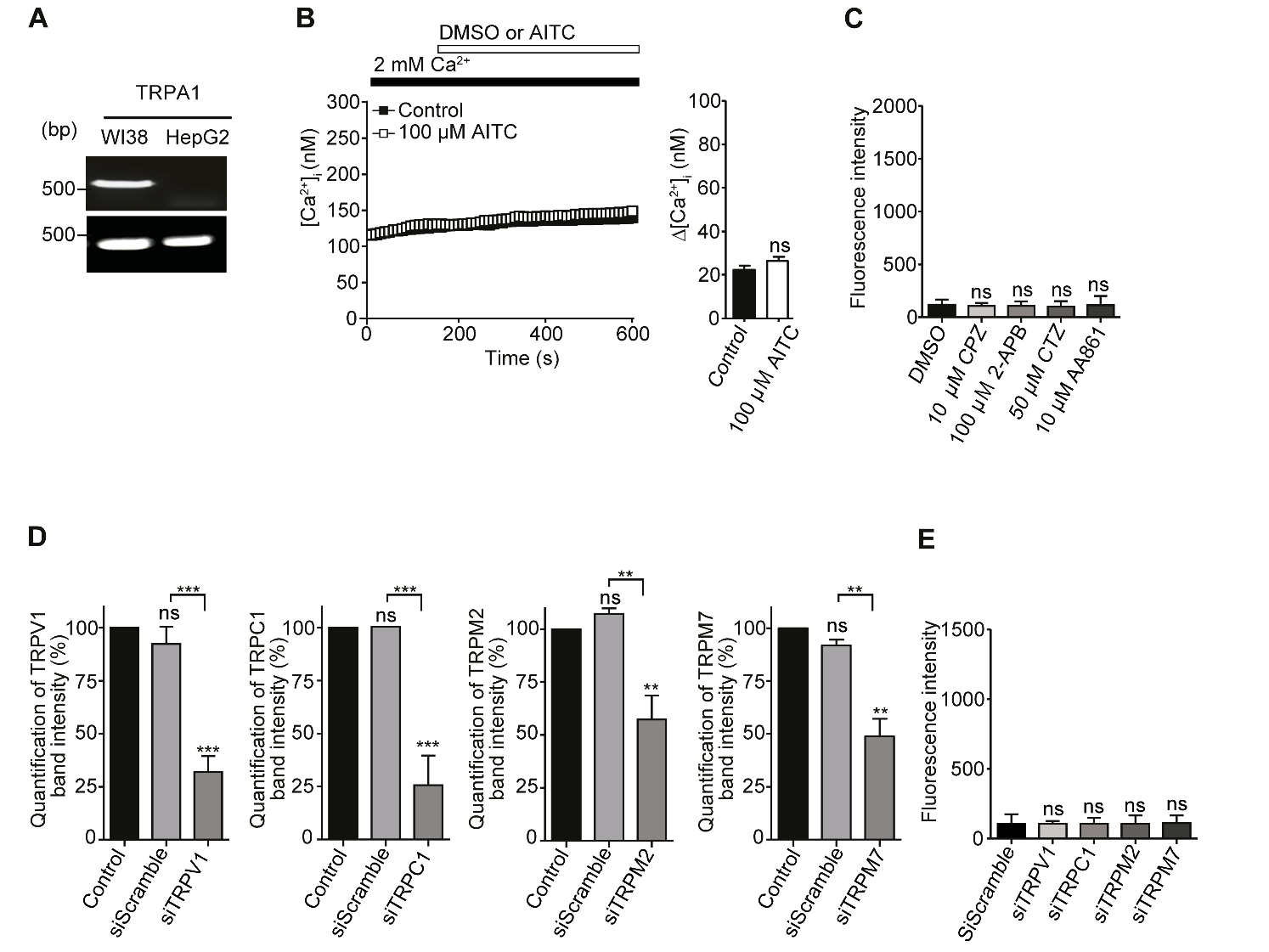


Supplementary Figure 1


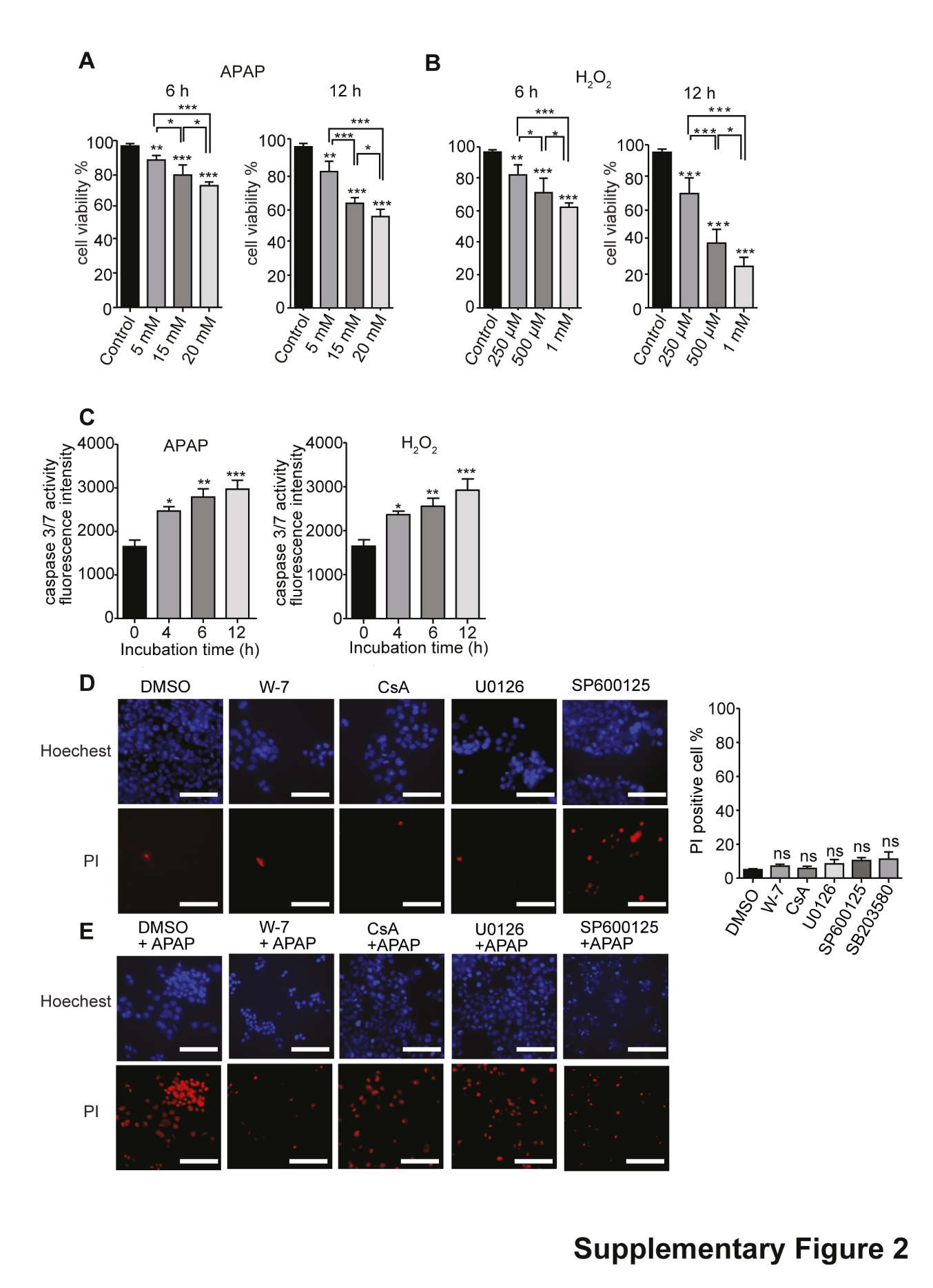


Supplementary Figure 2


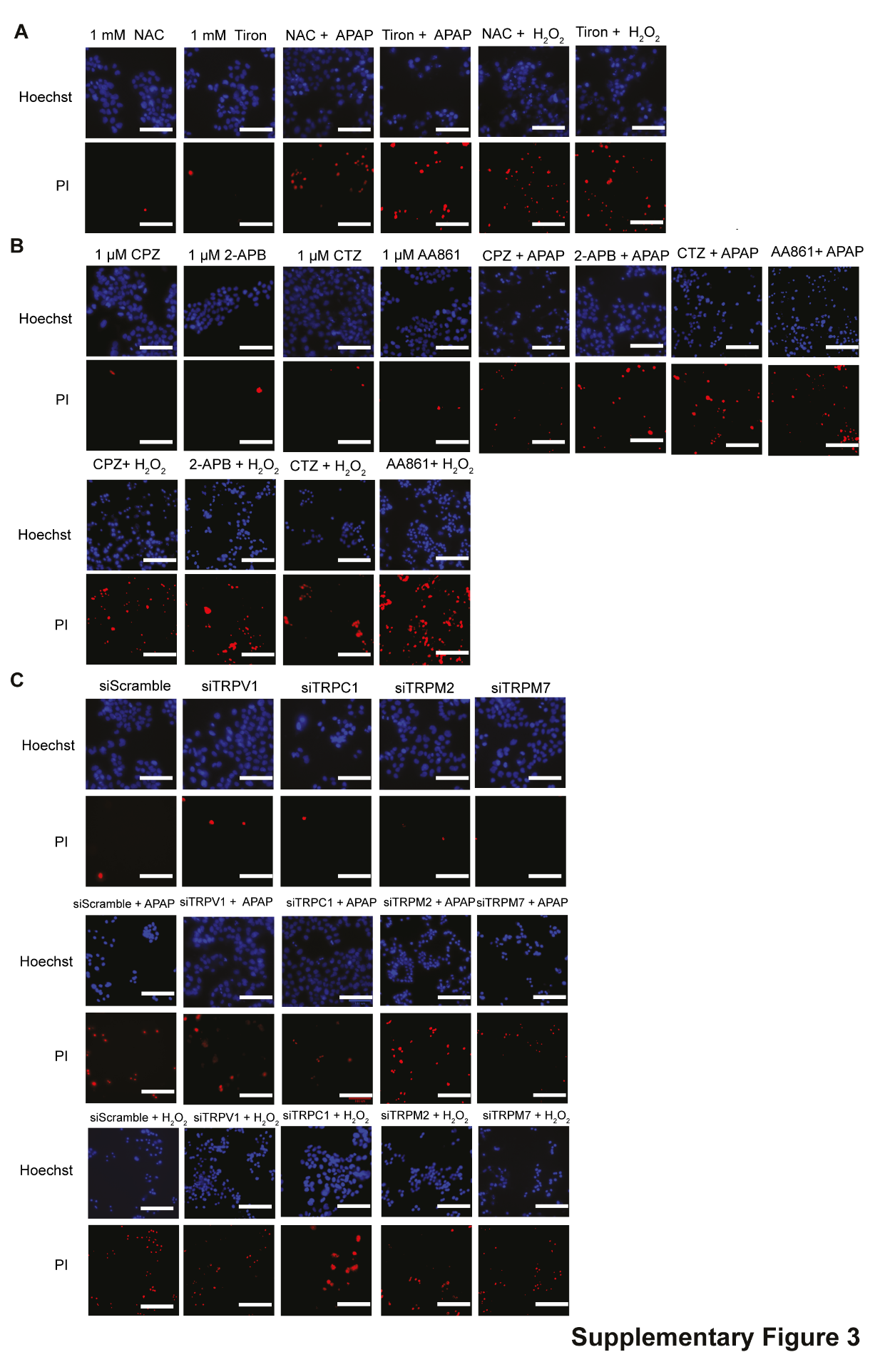


Supplementary Figure 3


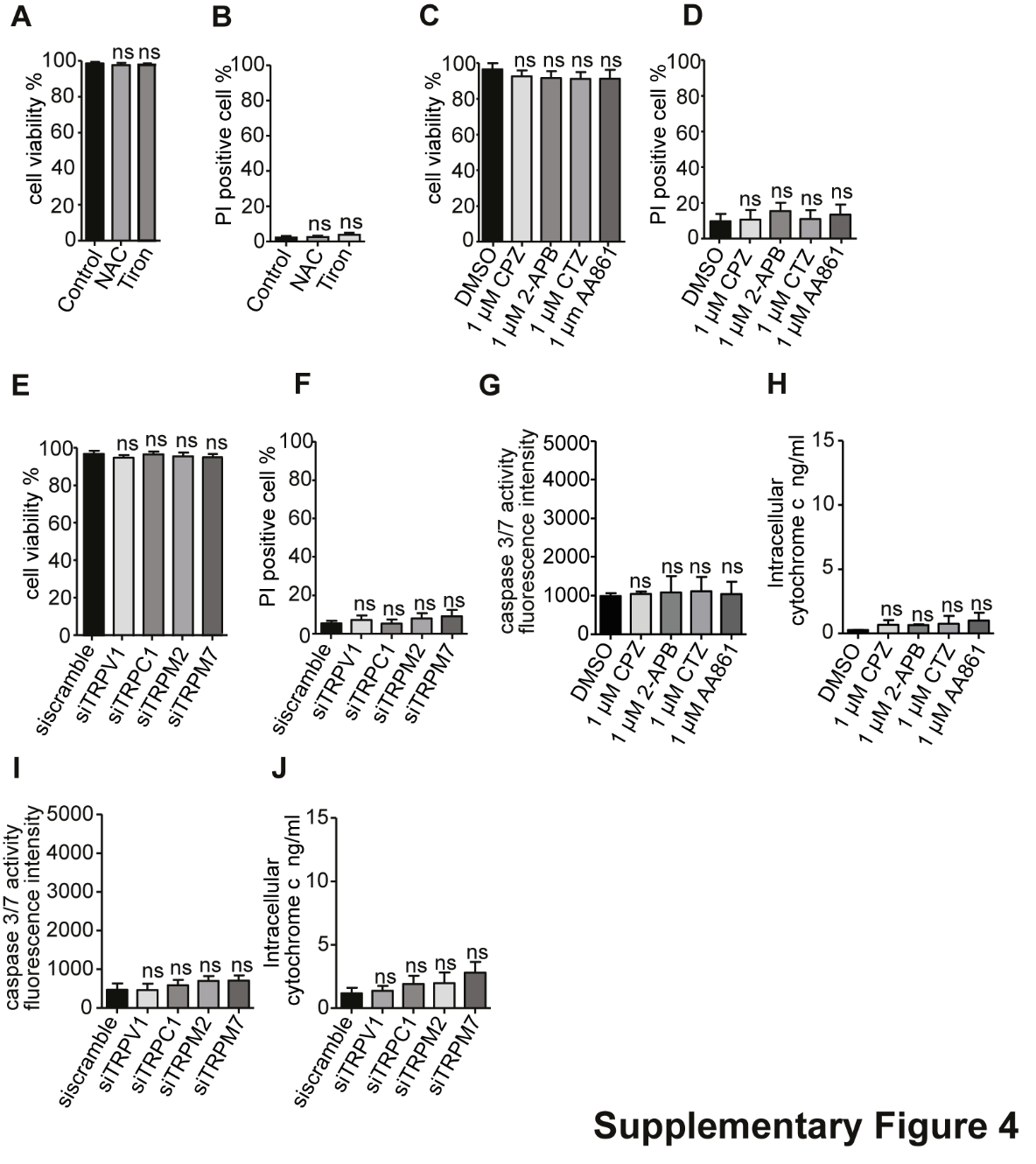


Supplementary Figure 4


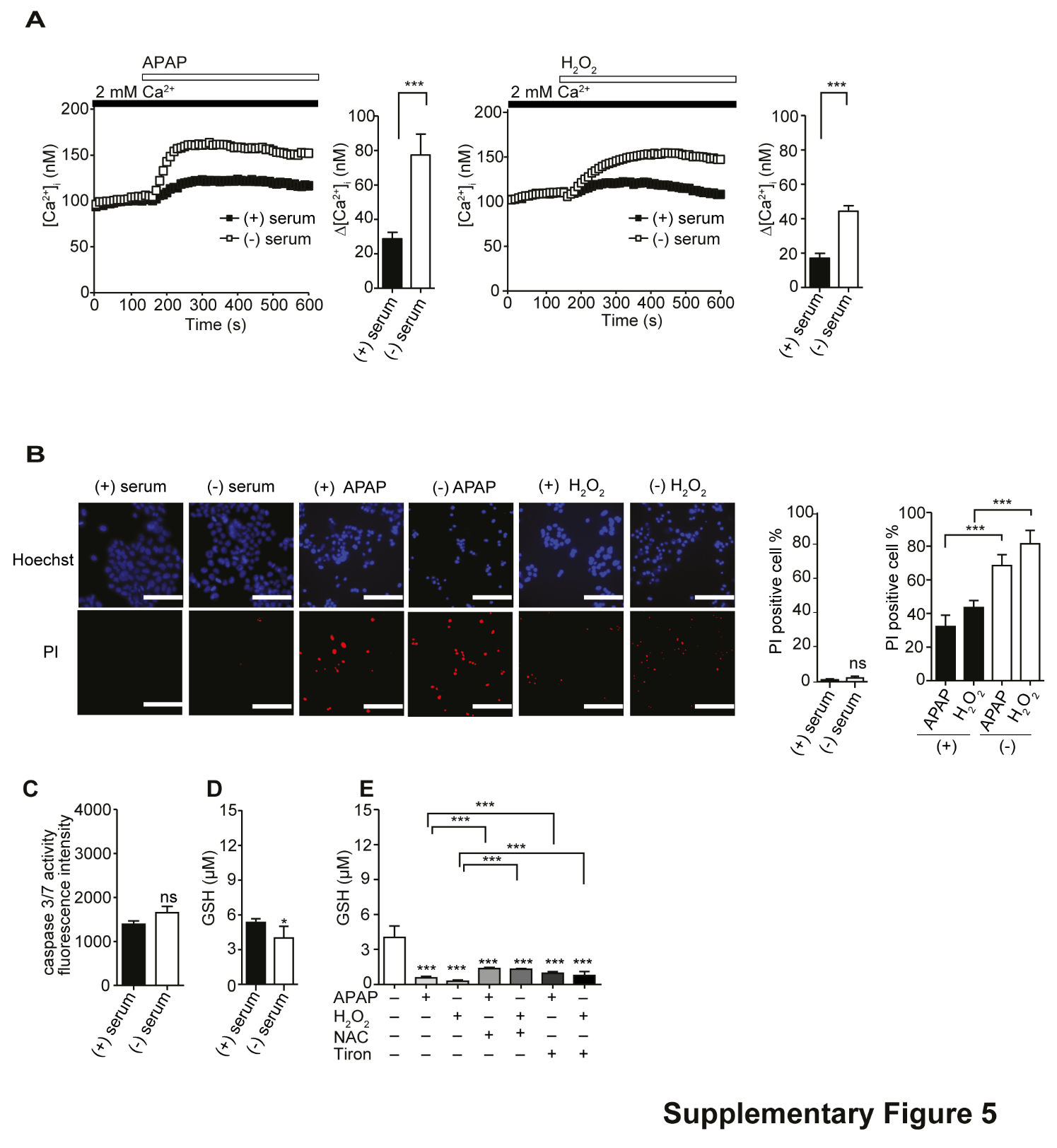


Supplementary Figure 5


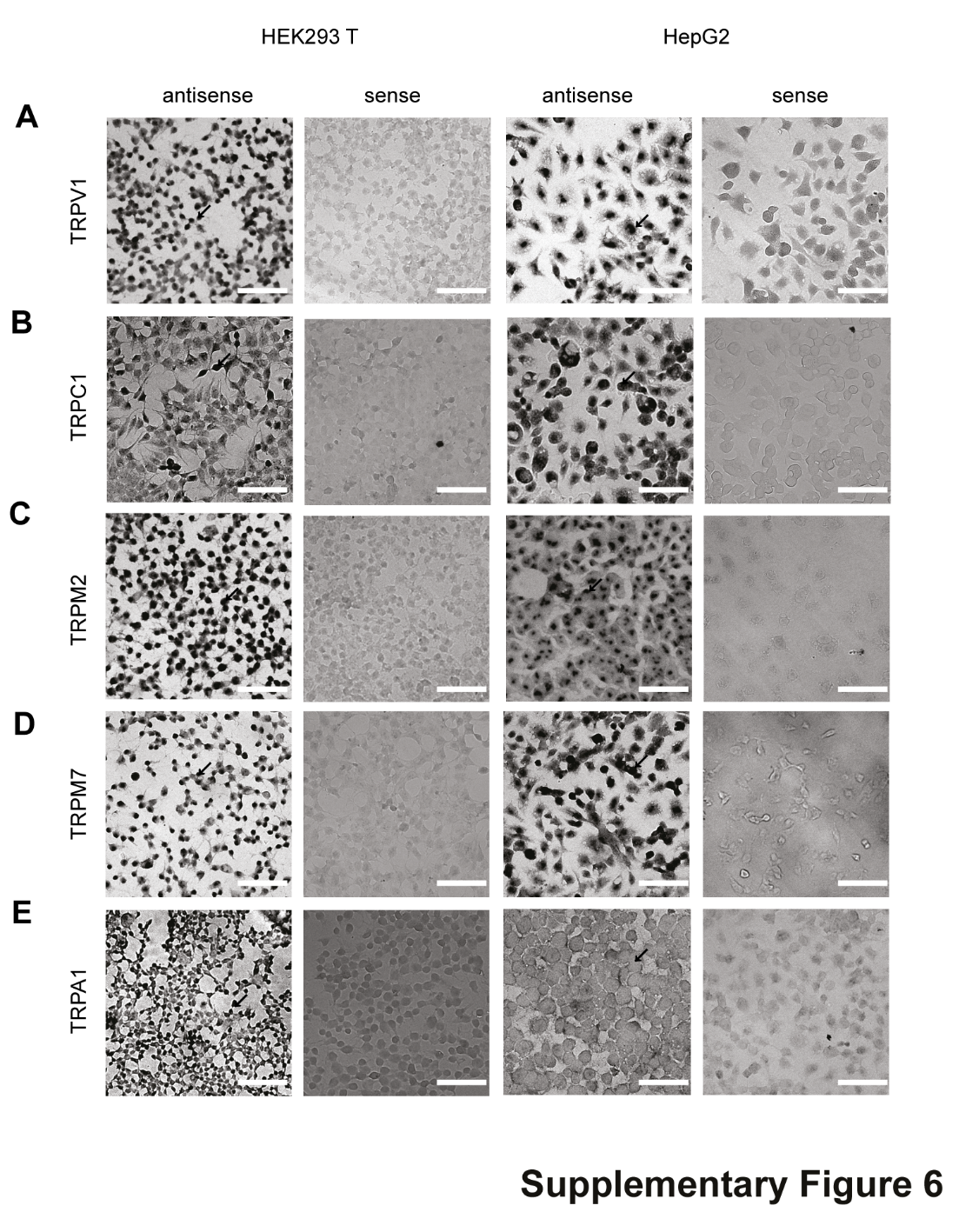


Supplementary Figure 6
